# RIPK1 deficiency prevents thymic NK1.1 expression and subsequent iNKT cell development

**DOI:** 10.3389/fimmu.2023.1103591

**Published:** 2023-10-30

**Authors:** Thomas Hägglöf, Raksha Parthasarathy, Nathaniel Liendo, Elizabeth A. Dudley, Elizabeth A. Leadbetter

**Affiliations:** ^1^ Department of Microbiology, Immunology & Molecular Genetics, University of Texas Health at San Antonio, San Antonio, TX, United States; ^2^ Laboratory of Molecular Immunology, The Rockefeller University, New York, NY, United States; ^3^ St Mary’s University, San Antonio, TX, United States

**Keywords:** RIPK1, caspase 8, RIPK3, iNKT cell development, NK1.1+ cells

## Abstract

Receptor Interacting Protein Kinase 1 (RIPK1) and caspase-8 (Casp8) jointly orchestrate apoptosis, a key mechanism for eliminating developing T cells which have autoreactive or improperly arranged T cell receptors. Mutations in the scaffolding domain of *Ripk1* gene have been identified in humans with autoinflammatory diseases like Cleavage Resistant RIPK1 Induced Autoinflammatory (CRIA) and Inflammatory Bowel Disease. RIPK1 protein also contributes to conventional T cell differentiation and peripheral T cell homeostasis through its scaffolding domain in a cell death independent context. *Ripk1* deficient mice do not survive beyond birth, so we have studied the function of this kinase *in vivo* against a backdrop *Ripk3* and *Casp8* deficiency which allows the mice to survive to adulthood. These studies reveal a key role for RIPK1 in mediating NK1.1 expression, including on thymic iNKT cells, which is a key requirement for thymic stage 2 to stage 3 transition as well as iNKT cell precursor development. These results are consistent with RIPK1 mediating responses to TcR engagement, which influence NK1.1 expression and iNKT cell thymic development. We also used *in vivo* and *in vitro* stimulation assays to confirm a role for both Casp8 and RIPK1 in mediating iNKT cytokine effector responses. Finally, we also noted expanded and hyperactivated iNKT follicular helper (iNKT_FH_) cells in both DKO (*Casp8-, Ripk3-* deficient) and TKO mice (*Ripk1-, Casp8-, Ripk3-* deficient). Thus, while RIPK1 and Casp8 jointly facilitate iNKT effector function, RIPK1 uniquely influenced thymic iNKT cell development most likely by regulating molecular responses to T cell receptor engagement. iNKT developmental and functional aberrances were not evident in mice expressing a kinase-dead version of RIPK1 (RIPK1kd), indicating that the scaffolding function of this protein exerts the critical regulation of iNKT cells. Our findings suggest that small molecule inhibitors of RIPK1 could be used to regulate iNKT cell development and effector function to alleviate autoinflammatory conditions in humans.

## Introduction

Recent studies on a cohort of patients with a newly-described cluster of autoimmune symptoms (CRIA), characterized by early onset fevers and lymphadenopathy, were found to have common underlying gain-of-function mutations which rendered Receptor-Interacting Serine/Threonine-Protein Kinase 1 (RIPK1) protein resistant to caspase 8 (Casp8) cleavage. Patients also had increased inflammatory RNA signatures, increased TNF and IL-1β serum cytokines, and hyper-responsiveness to TLR ligands. RIPK1^mut/+^ mice with a similar mutation were also hyper-responsive to TLR activation, but neither mice or human patients showed effects on TNF-induced NF-κB or MAPK. Instead, CRIA patients responded to an IL-6 inhibitor (tocilizumab), but not to TNF inhibitors, suggesting that in these patients and their murine equivalents, RIPK1 mediates a role beyond TNF-signaling in key lymphocyte populations (1). CRIA patients suffer from early-onset recurrent fevers and lymphadenopathy in addition to clusters of other symptoms, but the specific immunodeficiencies underlying the disease remain undescribed. The role of RIP kinases in cell death pathways are well known; RIPK3 kinase mediates necroptosis, Casp8 enables apoptosis, and RIPK1 facilitates pro-survival signals through NF-κB while also orchestrating necroptosis and apoptosis (2).

Mice with exclusive RIPK1 deficiency do not survive long past birth and mice lacking Casp8 do not survive embryogenesis, which has complicated attempts to study the exclusive roles of these kinases in immune responses (3–5). However, genetically engineering mice to lack both RIPK3 and Casp8 (DKO: RIPK3^-/-^, Casp8^-/-^) rescues these mice and allows them to survive (4, 6). RIPK1, Casp8, and RIPK3 triple deficient mice (TKO: RIPK1^-/-^, RIPK3^-/-^, casp8^-/-^) also survive well into adulthood (7). Both the DKO and TKO mice present with autoimmune-like features including splenomegaly, lymphadenopathy, and the presence of an autoinflammatory B220^+^ CD3^+^ cell population which accumulates with age, reminiscent of Fas/FasL deficient mice, which likely contributes to the Autoimmune Lymphoproliferative Syndrome (ALPS) in these mice (7, 8). These DKO and TKO mouse strains also accumulate germinal center B cells, CD4 T cells, and various subsets of CD8 T cells (4, 7). RIPK1 kinase “dead” (RIPK1kd) mice bear a K45A mutation in the kinase domain abrogating the kinase function but retaining complete scaffold function (7), thereby providing a means to differentiate between the kinase and scaffold functions of RIPK1. Cleavage of RIPK1 by Casp8 negatively regulates RIPK1 function and limits apoptosis and necroptosis (9), so mice lacking Casp8 have unregulated activity of RIPK1. Therefore, in the DKO mice, the RIPK1 molecule is most likely overactive (4, 10). However, in the TKO mice, RIPK1 activity is completely absent (7). This allows a comparison of DKO mice, which express constitutively active RIPK1, to TKO mice, which lack RIPK1, as a means to assess physiological functions of RIPK1.

We have also recently shown that mice differentially expressing RIPK1 against a background of *Casp8* and *Ripk3* deficiency had differences in innate B cell population sizes, including B-1 and MZB cells, suggesting these populations depend on RIPK1 expression (11). Indeed, previous studies characterized a role for RIPK1 in mediating signals downstream of T cell receptors (TcRs). First, despite normal development at 4 weeks, few mature T cells were sustained when immuno-deficient mice are reconstituted with RIPK1-deficient fetal liver cells (12, 13), suggesting RIPK1 signals are critical for T cell development. More specifically, RIPK1 is induced in thymocytes following positive selection, a step mediated by TcR recognition of MHC, but survival of those thymocytes requires that cell-death activity of RIPK1 be repressed by IKK (14). In this way, RIPK1 mediates survival of thymic precursors which have received engagement of both TcR and TNFR. Mice with T cell-restricted deletion of *Ripk1* or inactivated RIPK1 kinase domains have severe T cell development defects and secondary peripheral lymphopenia (15). T cells from these mice proliferated poorly in response to TcR engagement and have defective TcR-induced p65 NF-kB phosphorylation (16) consistent with a defect in TcR signaling. This places RIPK1 as a possible rheostat downstream of TcR signaling, mediating T cell selection, survival, and death in the thymus.

Given the CRIA syndromes which implicate RIP kinases in autoimmunity, the data described above, and as a corollary to our recent publication which established a role for RIPK1 in innate B cell development (11), we hypothesize that RIPK1 functions as a rheostat to relay TcR signal strength which influences innate T cell development and function. In this study, we examined the role of RIPK1 in innate T cell development and peripheral effector function.

Indeed, our data show that iNKT cell development, and, in part, peripheral pro-inflammatory effector function are dependent on RIPK1. Peripheral iNKT cell frequency diminishes in the absence of RIPK1/Casp8, but MAIT cells and gdT cells were unaffected. We also showed that RIPK1 deficiency stalls iNKT development at stage 2. Similarly, iNKT precursor (iNKTp) cells accumulate in the thymus in the absence of RIPK1. The accumulation of stage 2 iNKT cells and iNKTp is likely to be a consequence of the failure of iNKT cells to express NK1.1 in the absence of RIPK1. Splenic iNKT cells from DKO and TKO mice responded poorly to immunization with an iNKT agonist, αGalactosylCeramide (αGC), and had reduced IFNγ and IL-17 production, implicating a role for Casp8 in effector cytokine responses. iNKT_FH_ cells are expanded and possess an activated phenotype in naïve mice lacking RIPK1/Casp8, which does not change upon immunization. Importantly, iNKT developmental and functional aberrances were not evident in mice expressing a kinase-dead version of RIPK1 (RIPK1kd), indicating that the scaffolding function of this protein exerts the critical regulation of iNKT cells. Only Nur77 expression was decreased in RIPK1kd mice. These data suggest that RIPK1 may be required for the transduction of TcR signals in iNKT cells. The impaired TcR signal hinders iNKT cell development, inflammatory cytokine effector function, and adaptive help. Therefore, our data suggest that RIPK1 acts downstream of the semi-invariant αβTCR and has a rheostat like function in mediating TcR signal transduction.

## Materials and methods

### Mice

RIPK3-/-, DKO, TKO and RIPK1kd mice were a kind gift from Dr. William Kaiser (UTHSCSA) (4, 7). C57BL/6, B6.129 S4 IFNγ tn3.Ilky (“GREAT” mice which report IFNγ with cytoplasmic production of eYFP) (17) and B6.KN2 Cre mice which report IL-4 production by surface expression of the human CD2 protein (18) (detected by anti-human CD2 antibodies in flow cytometry) were housed and bred under specific pathogen-free (SPF) conditions and all mice were maintained at the Laboratory Animal Resources (LAR) animal facility of UT Health San Antonio. All experiments were performed using IACUC approved protocols. Age- and sex-matched female or male mice were used in all experiments, primarily aged 6-14 weeks, with a few experiments using mice up to 28 weeks, as noted in the legends.

### H&E staining

6μm-8μm sections of optimal cutting temperature (OCT compound, Fisher Healthcare)-embedded murine thymi were cut using a cryostat NX (Thermo Scientific) and fixed in 95% alcohol formalin. Tissue was stained with hematoxylin and eosin, washed with 95% alcohol, and coverslipped using Cytoseal. 3-5 images per section, multiple sections per thymus, from multiple animals were imaged with a Zeiss microscope as noted in figure legends.

### Flow cytometry

3x10^6^ murine splenocytes or peritoneal washout cells per sample were treated with ACK lysis buffer (Lonza), non-specific labeling was blocked with anti-mouse CD16/32 (clone 93), and dead cells were excluded by staining with Live/Dead Fixable Zombie viability dye (BD Biosciences). Further surface labeling was performed using antibodies (BD Pharmingen, Biolegend, R&D Systems, or eBiosciences) outlined in **Supplementary Table 1**.

Doublet exclusion and live-cell discrimination was performed on all cell populations prior to sub-gating. Samples were acquired on a BD FACS Celesta (Becton Dickinson) and Cytek Aurora (Cytek) at the UT Health San Antonio flow cytometry core and analyzed using FlowJo software (10.5.3). CD1d-PBS57 and 5-OP-RU-loaded MR1 tetramers were provided by the NIH Tetramer Core Facility. Cell numbers of certain lymphocyte populations were determined by counting total leukocytes per whole organ using trypan blue diluted single cell suspensions on a hemocytometer. Total organ cell numbers were combined with flow cytometry-derived percentages of live cells, and percentages of serially gated cell populations of interest to calculate absolute numbers of each relevant population.

### OMIQ

OMIQ cytometry analysis software was used for dimensionality reduction analysis. Dimensionality reduction using UMAPs was performed by excluding doublets and dead cells. iNKT_FH_ cells were then identified as CD1d tet^+^, TCRβ^int^, CD4^+^, CXCR5^+^, and PD-1^+^. UMAPs of splenic iNKT_FH_ cells from naïve and immunized mice (NP-KLH+αGC or NPαGC) used mean fluorescent intensity parameters of ICOS, PD-1, Tim3, and Lag3 with default settings.

### RNA isolation

RNA was isolated according to manufacturer’s instructions (Direct-zol RNA microprep; Catalog #R2062). In short, splenocytes and thymocytes were lysed in QIAZol buffer. A volume of 95-100% ethanol equal to the volume of QIAZol was added and the mixture was transferred into a zymo spin column. After a few wash steps, DNase treatment was performed to remove genomic DNA. RNA was then eluted in nuclease free water.

### cDNA synthesis and PCR

cDNA was synthesized using the MultiScribe cDNA synthesis kit according to manufacturer’s instruction. Up to 2μg of RNA was reverse transcribed in a buffer mixture consisting of dNTPs, MultiScribe Reverse Transcriptase, Random Primers, and RNase inhibitor. cDNA was used to amplify NK1.1 mRNA transcripts, specifically NKRP1A, NKRP1B and NKRP1C. PCR master mix consisted of dNTPs, PCR buffer, MgCl_2_, Taq Polymerase with appropriate forward primers and reverse primers outlined in **Supplementary Table 2**. Polymerase Chain Reaction) protocol is as follows: 25°C for 10 mins -> 37°C for 120 mins -> 85°C for 5 mins. PCR products were resolved on a 1.5% agarose gel containing 1% Gel Green (Biotium, Catalog #: 41004 - 41005-T) and imaged using a BioRad GelDoc system.

### Immunizations

To assess cytokine production, WT, RIPK3^-/-^, DKO, TKO, and RIPK1kd mice were immunized intravenously (IV) with 0.5μg of αGalactosylCeramide (αGC; Avanti Polar Lipids) in 200μL of PBS + 0.1% BSA and organs harvested 4 hours later for flow cytometry assessment. To measure iNKT_FH_ responses, WT, RIPK3^-/-^, DKO, TKO and RIPK1kd mice were immunized IV with 0.5μg/μL NP-KLH and 0.5μg/200μL αGC or 0.5μg/200μL of NPαGC and organs harvested 5 days later.

### *In-vitro* stimulations

2x10^6^ splenocytes from B6.129 S4 IFNγ tn3.Ilky (“GREAT” mice IFNγ eYFP reporter) mice or B6.KN2 Cre mice were pretreated *in vitro* with 39.5μM RIPK inhibitor, Necrostatin-1 (Cat# N9037, Sigma-Aldrich) for 60 minutes. Control untreated and pretreated cells were then stimulated with various concentrations of αGC (10ng/mL, 50ng/mL and 100ng/mL). To observe peak cytokine responses, splenocytes from GREAT mice and B6.KN2 mice were analyzed by flow cytometry 4 and 12 hours after stimulation, respectively. PMA/Ionomycin (2μg/mL), αCD3 (50μg/mL), and αCD28 (25μg/mL) were used as positive controls.

### Statistical analysis

Statistical significance was assessed by GraphPad PRISM 7 using a 1-way ANOVA as indicated. Variance is similar between all groups compared, and data points were excluded from consideration if identified by the ROUT method using Graphpad PRISM software. Box heights and circle, triangle, box centers indicate group means and/or individual values. Error bars indicate s.e.m.

## Results

### Casp8 is required for innate iNKT cell homeostasis

To study the *in vivo* role of RIPK1 in innate T cell homeostasis, we used flow cytometry to quantify three innate T cell populations and their distribution in secondary lymphoid organs in WT, RIPK3^-/-^, DKO, TKO, and RIPK1kd mice (**Supplementary Figure 1**). Because of their large likely-autoimmune CD3^+^B220^+^ population, DKO mice had a significantly lower frequency of total T cells (TCRβ^+^ T cells) in the spleen when compared to WT mice and mice of other genotypes (**Supplementary Figure 1A**). However, the absolute number of total T cells in the spleen of DKO and TKO mice was comparable to RIPK3^-/-^, RIPK1kd, and control WT mice (**Supplementary Figure 1B**). Nevertheless, to minimize distortion of frequencies of lymphocyte populations, the autoimmune CD3^+^ B220^+^ cells were excluded from analysis during flow cytometry gating.

**Figure 1:**
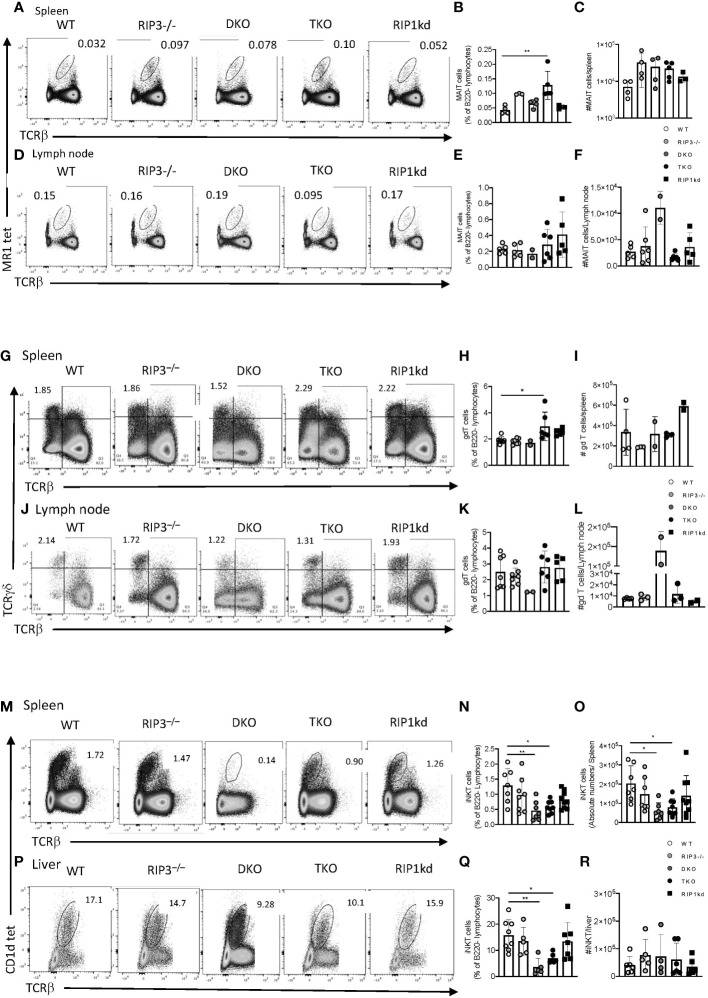
Casp8 deficiency reduces iNKT cell but not MAIT or γδT cell frequency and number in the periphery. Flow cytometry analysis of WT, RIPK3^-/-^, DKO, TKO and RIPK1kd mice identifies MR1 tet^+^ TCRβ^int^ MAIT cells in the spleen **(A–C)** and inguinal lymph node **(D–F)** as a percentage of B220^-^ live lymphocytes. Scatter plot quantifies frequency **(B, E)** and number **(C, F)** of MAIT cells in spleen **(B, C)** and inguinal LN **(E, F)**. Flow cytometry also identified (live B220^-^ TCRγδ^+^ TCRβ^-^) γδ^+^T cells in spleen **(G–I)** and inguinal LN **(J–L)**. Scatter plots quantify frequency **(H, K)** and number **(I, L)** of γδ^+^T cells in the spleen **(H, I)** and inguinal lymph node **(K, L)**. Finally, flow cytometry identified (B220^-^CD1d tet^+^TCRβ^int^) iNKT cells in the spleen **(M–O)** and inguinal LN **(P–R)**. Scatter plots quantify frequencies **(N, Q)** and number **(O, R)** of iNKT cells in the spleen **(N, O)** and liver **(Q, R)**. Data is representative **(A, D, G, J, M, P)** or a pool **(B, C, E, F, H, I, K, L, N, O, Q, R)** of 3-4 independent experiments with 2-8 mice per group, 8-16 weeks old. Each symbol indicates an individual mouse. *p ≤ 0.05, **p ≤ 0.01 (One-way ANOVA).

To determine the impact of RIPK1 deficiency on innate immune T cells, we first looked at the distribution and frequency of innate-like Mucosal Associated Invariant T (MAIT) cells in the secondary lymphoid organs by flow cytometry (gating strategy in **Supplementary Figure 1E, F**). TKO mice had a significantly expanded frequency of splenic MAIT cells, identified using the 5-OP-RU loaded MR1 tetramer, compared to WT controls (**Figures 1A, B**), but this difference did not extend so far as to be a specific increase in MAIT cell number (**Figure 1C**). We also did not observe any significant difference in the frequency or cell number of MAIT cells in the lymph nodes from WT, RIPK3-/-, TKO, and RIPK1kd mice (**Figures 1D–F**). Surprisingly, although there were no differences in frequency, DKO mice did show an isolated expansion of MAIT cell numbers in LNs (**Figure 1F**). In short, alterations in MAIT cell frequencies and numbers in DKO or TKO mice do not seem to reflect any consistent trends. Next, we examined another innate T cell population, γδT cells, in mice lacking Casp8 and/or RIPK1. Using flow cytometry (gating strategy in **Supplementary Figure 1G, H**), we found that TKO mice had a significantly higher frequency of γδT cells in the spleen than WT and trending differences from all other controls but again this did not extend to include changes in splenic γδT cell numbers (**Figures 1G–I**). Similarly, γδT cell frequencies and numbers in the lymph nodes of DKO and TKO mice did not significantly differ from WT mice or other controls (**Figures 1J–L**). These isolated increases in frequencies (but not numbers) of MAIT and γδT cells in the spleens of TKO mice most likely reflect losses of other peripheral populations, and do not support a general RIPK1-dependent defect in these two innate T cell populations.

We next used the PBS57-loaded CD1d tetramer to identify iNKT cells in peripheral organs; spleen, and liver (**Figures 1M-R**) (gating strategy in **Supplementary Figure 1I, J**). An unloaded CD1d tetramer was used to determine gating. DKO and TKO mice exhibited significantly lower frequencies and numbers of splenic iNKT cells compared to WT, RIPK3^-/-^, and RIPK1kd mice (**Figures 1N, O**). To confirm that the changes in frequency of iNKT cells in the spleen were not simply a reflection of overall impaired proliferation or cell death, we measured the frequency and number of total T cells, as well as the frequency of dead lymphocytes in the spleen (**Supplementary Figure 1**). There was a modest drop in total splenic T cell frequency in DKO mice (**Supplementary Figure 1A**), but there were no changes in the number of total splenic T cells across all genotypes of mice tested (**Supplementary Figure 1B**). There were also no differences in percentage of dead lymphocytes in the spleen of any strains tested, but DKO mice had a modest increased in the number of dead lymphocytes in the spleen when compared to all other genotypes tested (**Supplementary Figure 1C, D**). The liver, a major reservoir for iNKT cells, mirrored the spleen with a significantly lower frequency of iNKT cells in DKO and TKO compared to WT (**Figure 1P, Q**), but the changes in hepatic iNKT frequency were not reflected by changes in cell numbers (**Figure 1R**). This suggests the iNKT changes in the liver reflected alterations in another cell population, and not a direct effect on iNKT cells themselves. In summary, since both DKO and TKO mice display a lower frequency and number of iNKT cells in the liver and/or spleen, these effects are consistent with a role for Casp8 (deficient in both DKO and TKO) in mediating iNKT cell development or peripheral persistence.

### RIPK1 and Casp8 deficiency does not disrupt peripheral iNKT subsets

**Figure 2:**
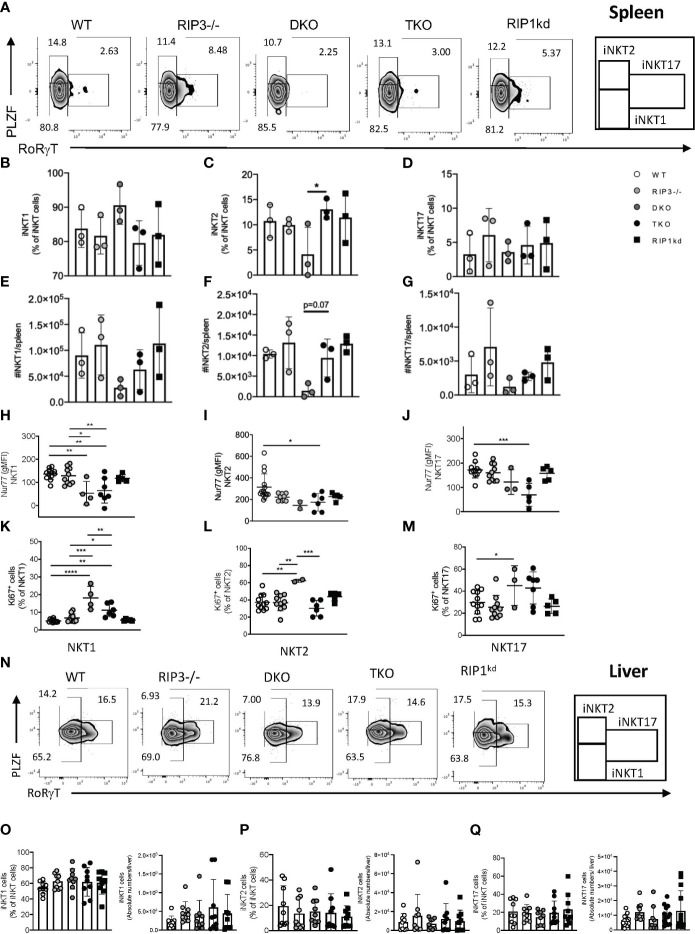
Splenic iNKT1, iNKT2, iNKT17 subset frequency and number depend on Casp8. Representative flow cytometry plots identifying splenic iNKT1 (PLZF^lo^, RoRγT^-^), iNKT2 (PLZF^hi^, RoRγT^-^) and iNKT17 (PLZF^int^, RoRγT^+^) cell frequencies as percent of iNKT (CD1d tet+, TCRβ^int^, B220-) cells in the spleen of WT, RIPK3^-/-^, DKO, TKO and RIPK1kd mice **(A)**. Scatter plot quantifies frequency **(B–D)** and number **(E–G)** of iNKT1, iNKT2, and iNKT17 cells. Scatter plots quantify gMFI of Nur77 in splenic iNKT1 **(H)**, iNKT2 **(I)** and iNKT17 **(J)** cells. Scatter plot quantifies frequency of Ki67+ iNKT1 **(K)**, iNKT2 **(L)** and iNKT17 **(M)** cells. Flow cytometry plots identifying iNKT1 (PLZF^lo^, RoRγT^-^), iNKT2 (PLZF^hi^, RoRγT^-^) and iNKT17 (PLZF^int^, RoRγT^+^) cell frequencies as percent of iNKT (CD1d tet+, TCRβ^int^, B220-) cells in the liver of WT, RIPK3^-/-^, DKO, TKO and RIPK1kd mice **(N)**. Scatterplot quantifies frequency and number of iNKT1 **(O)**, iNKT2 **(P)**, and iNKT17 cells **(Q)**. Data is representative **(A,N)** or a pool **(B–M, O–Q)** of 3-4 independent experiments with 2-8 mice per group, 8-16 weeks old. Each symbol indicates an individual mouse. *p ≤ 0.05, **p ≤ 0.01, ***p ≤ 0.001, ****p ≤ 0.0001 (One-way ANOVA).

We next used flow cytometry to examine whether changes in iNKT cell numbers reflected preferential alterations of certain iNKT cell subsets in the periphery and spleen of DKO, TKO (gating strategies in **Supplementary Figure 2A**). We did not observe any significant differences in iNKT1, iNKT2 or iNKT17 frequencies or number in spleen of TKO mice compared to WT controls (**Figures 2A–G**). On the other hand, DKO mice had a significantly lower frequency and moderately lower number of iNKT2 cells compared to the same population in TKO mice (**Figures 2C, F**)**.** In fact, DKO mice had detectable reductions in the number of all subsets of iNKT cells (NKT1, NKT2, and NK17), but these changes did not reach significance. However, these generalized iNKT cell losses in the DKO mice are consistent with the trend towards increased cell death evident in all splenic T cells in DKO mice and suggest the absence of RIPK1 (missing in TKO but not DKO) may rescue this downward trend in the TKO mice (**Supplementary Figure 1B**).

Nur77 expression is widely used as a surrogate for TcR signaling in T cells (19) and is necessary for the development and differentiation of iNKT cells into phenotypic subsets. Therefore, we next measured Nur77 expression in splenic iNKT subsets. Nur77 expression was significantly reduced in all splenic iNKT subsets from TKO mice when compared to WT mice and other genotypes (**Figures 2H–J**). There was a visible decrease in Nur77 in all iNKT subsets in DKO mice as well, but it only reached significance in the NKT1 subset (**Figures 2H–J**). To ensure that the observed changes in the Nur77 expression were not due to impaired proliferation of iNKT cells but a consequence of RIPK1/Casp8 deficiency, we measured Ki67 levels in splenic iNKT subsets from all mouse strains. In fact, Ki67 expression was increased in all three subsets of iNKT cells from DKO mice, suggesting lack of proliferation was not a factor in the loss of these iNKT cell subsets (**Figures 2K–M**).

To comprehensively assess the systemic requirements of RIPK1/Casp8 signaling to regulate iNKT cells, we analyzed the frequency and distribution of iNKT subsets in liver, another peripheral organ and a reservoir for iNKT cells (gating strategies in **Supplementary Figure 2B**). iNKT1, iNKT2 and iNKT17 cells did not significantly differ in frequency or number in the livers of all genotypes of mice analyzed (**Figures 2N–Q**), consistent with findings from the spleen. In conclusion, global RIPK3/Casp8 (in DKO) or RIPK1/Casp8/RIPK3 deficiencies (in TKO) do not skew splenic iNKT subset distribution. While the numbers of iNKT cells in spleens of DKO mice are visibly reduced, these changes are across all subsets and do not reach statistical significance. Thus, both DKO and TKO mice have relatively normal iNKT1, iNKT2, and iNKT17 frequency in the spleen. Thus, neither RIPK1 or Casp8 regulate peripheral iNKT subset frequency or distribution in the spleen and liver.

### Thymic maturation of iNKT cells is RIPK1-dependent

**Figure 3:**
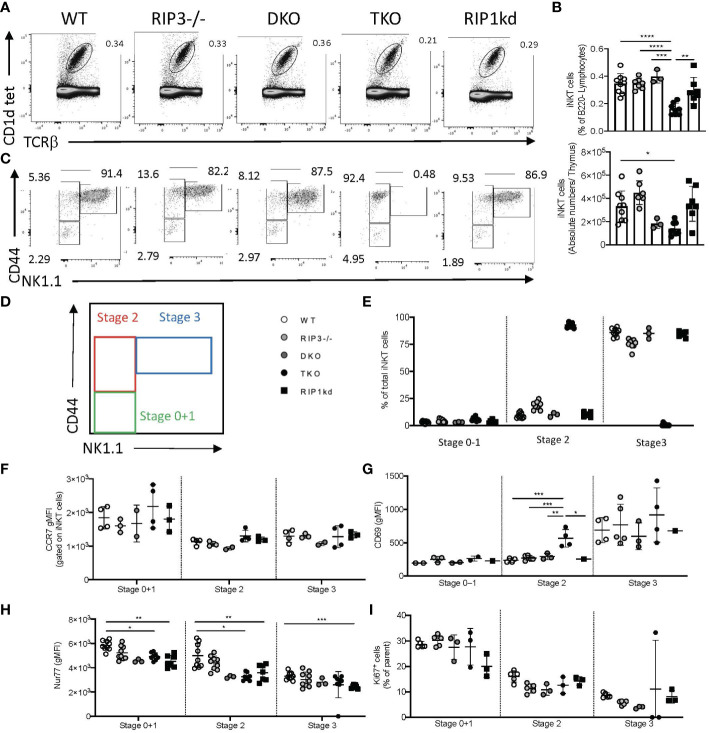
Thymic iNKT cells in TKO mice do not transition from stage 2 to stage 3. Representative flow cytometry plots capture thymic iNKT cells (CD1d tet^+^, TCRβ^int^) in WT, RIPK3^-/-^, DKO, TKO and RIPK1kd mice **(A)**. Scatter plot quantifies frequency (top) and number (bottom) of live B220^-^ thymic iNKT cells **(B)**. Representative flow cytometry identifies Stage 0 + 1 (CD44^-^ NK1.1^-^), stage 2 (CD44^+^ NK1.1^-^) and stage 3 (CD44^+^ NK1.1^+^) iNKT cells in the thymi of mice as indicated **(C, D)**. Scatter plot quantifies percentage of thymic iNKT cells at stage 0 + 1, stage 2, and stage 3 **(E)**. Quantifications also indicate CCR7 gMFI **(F)**, CD69 gMFI **(G)**, Nur77 gMFI **(H)**, and Ki67^+^ frequency of iNKT cells at noted stages of development in WT, RIPK3^-/-^, DKO, TKO and RIPK1kd mice. Data is representative **(A, C)** or a pool **(B, E–I)** of 2-4 experiments with at least 2-8 mice per group, 8-16 weeks old. Each symbol indicates an individual mouse. *p ≤ 0.05, **p ≤ 0.01, ***p ≤ 0.001, ****p ≤ 0.0001(One-way ANOVA).

The reduced frequency of total iNKT cells in the periphery of TKO mice could develop from a proliferation defect or a developmental defect. Since **Figures 1** and **2** have largely ruled out a proliferative defect, we next asked if reduction of total iNKT cells in the periphery of TKO mice could be a direct consequence of changes in iNKT development in the thymus. To this end, we used flow cytometry to identify and measure developing iNKT cell subsets in the thymus (gating strategies in **Supplementary Figure 3A**). TKO mice had a significantly reduced frequency and number of thymic iNKT cells when compared to all other genotypes (**Figures 3A, B**). We used CD44 and NK1.1 conventional markers to identify the iNKT developmental stages 0 + 1, 2, and 3 (**Figure 3C**). We did not observe any significant differences in the frequency of CD44^-^ and NK1.1^-^ stage 0 + 1 iNKT cells between any of the genotypes tested (**Figures 3C–E**). TKO mice had a significantly higher frequency of CD44^+^ NK1.1^-^ stage 2 iNKT cells than all other genotypes (**Figure 3C–E**). Concomitantly and surprisingly, TKO mice lacked virtually all CD44^+^ NK1.1^+^ stage 3 iNKT cells (**Figures 3C–E**). All other groups had normal WT levels of stage 3 iNKT cells (**Figure 3E**).

To determine if the accumulation of Stage 2 iNKT cells in the thymus of TKO mice reflects an impediment to thymic emigration, we measured CCR7 expression on thymic iNKT subsets (20). CCR7 expression was similar among all genotypes of mice during all stages of thymic iNKT development (**Figure 3F**). CD69 is another thymic emigration and egress marker for iNKT cells and is specifically required for the development of mature NKT2 cells (21). Therefore, we also measured the expression of CD69 on the surface of various developmental stages of thymic iNKT cells. Stage 0, 1 and 3 iNKT cells showed no differences in the expression of CD69 between any of the genotypes tested (**Figure 3G**). However, Stage 2 iNKT cells from the TKO mice expressed significantly higher levels of CD69 when compared to other genotypes (**Figure 3G**).

We next looked at intracellular Nur77 expression by Stage 0 + 1 and Stage 2 iNKT cells, since levels of Nur77 expression can serve as a surrogate for TcR engagement (22, 23). DKO, TKO, and RIPK1kd mice all had lower Nur77 expression compared to WT mice (**Figure 3H**), although only the changes observed in TKO and RIPK1kd mice reached significance. Differences in the RIPK1kd mice compared to WT mice are consistent with a possible role for the kinase domain in transducing TcR signals (**Figure 3H**) and could explain differences in the RIPK1-deficient TKO as well. We also measured Ki67 levels in all iNKT developmental stages to ensure that the changes we observed in numerous factors affecting iNKT cell development and egress were not due to impaired proliferation. Ki67 levels did not differ significantly in any iNKT developmental stages among all genotypes of mice tested (**Figure 3I**).

To consider a contributory role for thymic architectural changes in mediating the developmental defects observed in iNKT cells, we used H&E staining to assess the thymic architecture of WT, RIPK3^-/-^, DKO, TKO and RIPK1kd mice. We observed no visible architectural changes in the thymic cortex or medulla (**Supplementary Figure 3B**).

**Figure 4:**
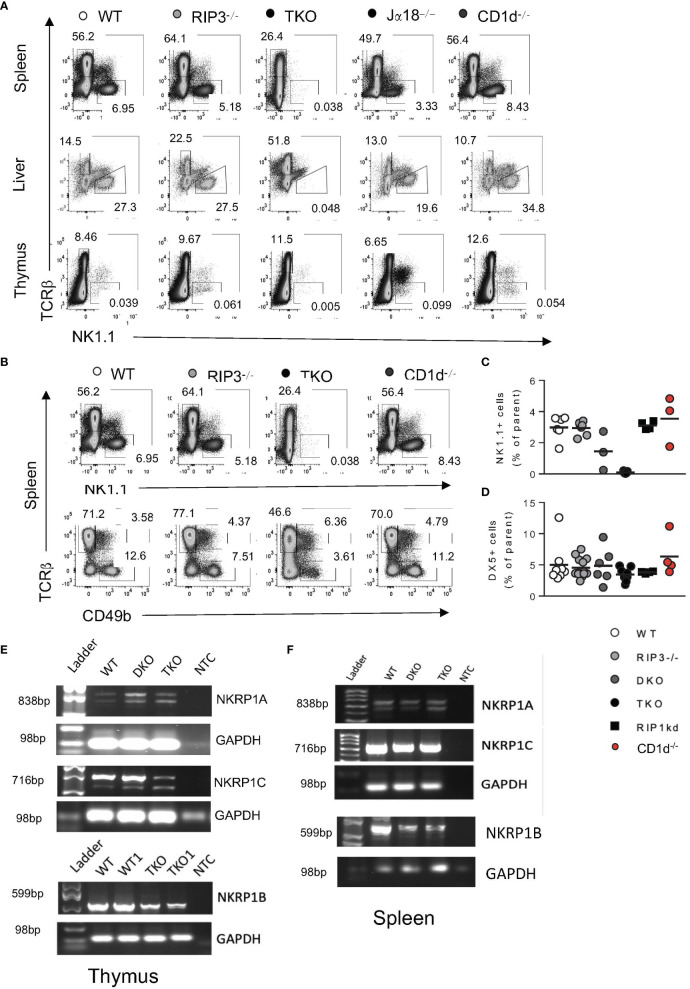
TKO mice have reduced NK1.1 protein expression in thymus and spleen. Representative flow plots depicting TcRβ and NK1.1 expression by cells in the spleen, liver and thymus of WT, RIPK3-/-, TKO, Jα18-/-, and CD1d-/- mice **(A)**. Representative flow plots capturing splenic NK cells as NK1.1^+^, TCRβ^-^ **(B)**, top) or as CD49b (DX5^+^TCRβ^-^) **(B)**, bottom) in WT, RIPK3-/-, TKO, and CD1d-/- mice. Scatter plots quantify NK1.1^+^ NK cells **(C)** and DX5^+^ NK cells **(D)** in WT, RIPK3-/-, DKO, TKO, RIP1kd, and CD1d-/- mice. Agarose gel images representing PCR products for *nkrp1a, nkrp1b* and *nkrp1c* or control GAPDH in the thymus **(E)** and spleen **(F)** of WT, DKO, and TKO mice as indicated. NTC= no template control. Data is representative **(A, B, E, F)** or a pool **(C, D)** or 2-4 independent experiments with 2-8 mice per group, 8-16 weeks old. Each symbol indicates an individual mouse.

Since NK1.1+ was the primary marker used to identify stage 3 iNKT cells, and it was completely absent in the thymus, we next examined NK1.1 expression on other cell types to determine if there was a general impairment in NK1.1 protein expression. Interestingly, NK1.1 protein expression was also profoundly lacking on TcRβ- cells, most likely NK cells, in the spleen, liver, and thymus of TKO mice as compared to WT mice (**Figure 4A**). Frequencies of DX5 (CD49b) cells were not significantly reduced in the spleens of TKO mice, suggesting RIPK1 deficiency and subsequent NK1.1 deficiency did not impact the development of NK cells as profoundly as iNKT cells (**Figures 4B–D**). We further confirmed the absence of NK1.1 protein by assessing the expression of NK1.1 mRNA in DKO and TKO thymus and spleen. Both the thymus and spleen of DKO and TKO mice express all isoforms of the NK1.1 mRNA (*nkrp1a, nkrp1b* and *nkrp1c*) in the spleen and the thymus (**Figures 4E, F**). However, expression of *nkrp1c*, the isoform specifically recognized by the anti-NK1.1 antibody clone PK136, is dramatically reduced in the thymus of TKO mice (**Figure 4E**). Levels of expression of mRNA from the *nkrp1b* isoform are more moderately reduced in both thymus and spleen of TKO mice (**Figures 4E, F**). Reductions in *nkrp1b* and *nkrp1c* are consistent with reductions detected in iNKT cell number in these organs (**Figure 4F**).

In summary, against a backdrop of RIPK3 and Casp8 deficiency, RIPK1 deficiency uniquely reduced total thymic iNKT cell frequency. An accumulation of NK1.1 negative stage 2 thymocytes only in TKO mice lacking RIPK1, but not DKO mice, also suggests RIPK1 is required for NK1.1 expression and stage 2 to stage 3 transition of developing iNKT cells. In the absence of RIPK1, we observed no changes in iNKT cell expression of CCR7 or Ki67, minimizing the possibility of migration or proliferation defects. However, reductions in intracellular Nur77 in thymocytes from TKO mice compared to controls suggests that reduced signal transduction downstream of the TcR may be contributing to the arrested development and accumulation of stage 2 iNKT cells in the TKO mice.

### RIPK1 regulates iNKT PLZF expression and is required for iNKT precursor homeostasis

**Figure 5:**
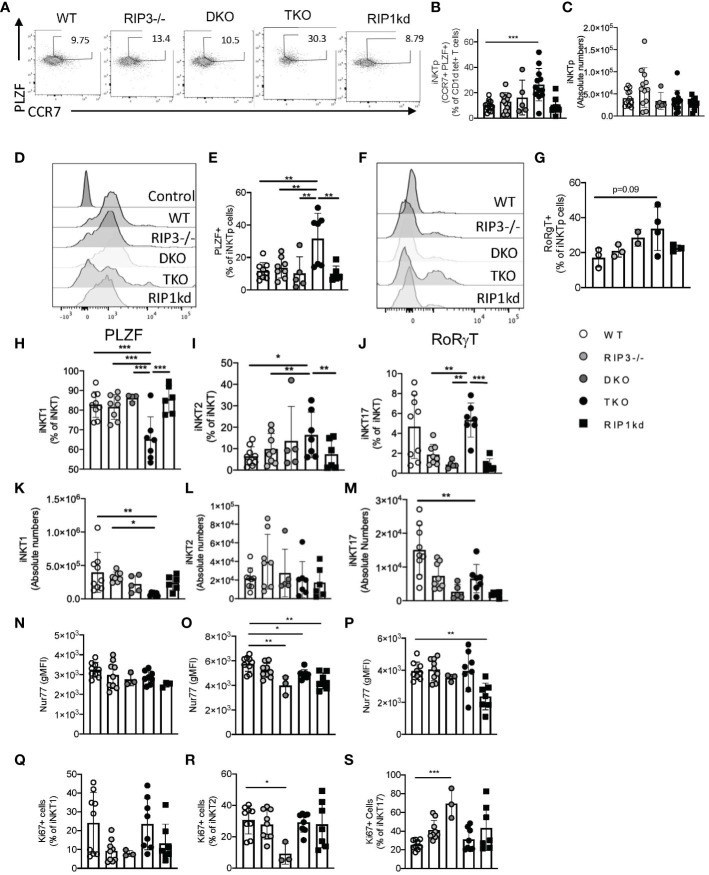
TKO mice develop increased frequency of thymic iNKTp cells. Representative flow cytometry plots reveal iNKTp (PLZF^hi^, CCR7^hi^, CD1d tet^+^, TCRβ^int^) populations in the thymi of WT, RIPK3^-/-^, DKO, TKO and RIPK1kd mice **(A)**. Scatter plot quantifies frequency **(B)** and number **(C)** of thymic iNKTp cells. Histograms represent PLZF **(D)** and RoRγT **(F)** expression levels in iNKTp cells. Scatter plot quantifies PLZF+ **(E)**, RoRγT+ **(G)** cells as a frequency of iNKTp cells. Scatterplots quantify thymic iNKT1 **(H)**, iNKT2 **(I)**, and iNKT17 **(J)** cells as a frequency of total iNKT cells. Scatterplot quantifies number of thymic iNKT1 **(K)**, iNKT2 **(L)**, and iNKT17 **(M)** cells. Scatterplot quantifies Nur77 expression as gMFI in thymic iNKT1 **(N)**, iNKT2 **(O)**, and iNKT17 **(P)** cells. Scatterplot quantifies Ki67+ cells as a frequency of thymic iNKT1 **(Q)**, iNKT2 **(R)**, and iNKT17 **(S)** cells. Data is representative **(A, D, F)** or a pool **(B, C, E, G, H–S)** of 2-3 experiments with 2-8 mice per group, 8-14 weeks old. Each symbol indicates an individual mouse. *p ≤ 0.05, **p ≤ 0.01, ***p ≤ 0.001 (One-way ANOVA).

Emerging research postulates that during development, iNKT cells attain a common precursor state that differentiates into the various effector subsets (24). iNKT precursors (iNKTp) express high levels of both PLZF and CCR7 (25). To determine the role of RIPK1 in iNKTp development and emigration, we measured the frequency of iNKTp cells in the thymus of mice lacking Casp8/RIPK3 (DKO) or Casp8/RIPK3 as well as RIPK1 (TKO). TKO mice had a significantly higher frequency of iNKTp when compared to all other genotypes (**Figures 5A, B**) (flow cytometry gating strategies in **Supplementary Figure 4A**). However, this change in frequency was not reflected as a change in iNKTp cell numbers for any mice tested (**Figure 5C**). Concomitantly, iNKTp cells from TKO mice expressed significantly higher levels of PLZF (MFI) compared to WT mice and DKO mice (**Figure 5D, E**). To determine if the absence of RIPK1 affects fate determining transcription factor expression in iNKTp cells, we measured RoRγT expression in iNKTp cells. RoRγT was expressed at similar low levels as determined by MFI in iNKTp cells across all genotypes tested except TKO mice (**Figures 5F, G**). A higher frequency of iNKTp cells from TKO mice were positive for RoRγT than WT controls but did not reach statistical significance (**Figures 5F, G**). Thus, in the absence of RIPK1 (in TKO mice), iNKTp cells accumulate in the thymus and trend towards increased RoRγT expression.

We next considered the effects of RIPK1 deficiency on individual thymic subsets, defining iNKT1, iNKT2, and iNKT17 cells by their relative expression of PLZF and RoRγT (**Supplementary Figure 4B**). Compared to WT and other controls, TKO mice had significantly reduced frequency of NKT1 cells, and concomitant increases in frequencies of NKT2 and NKT17 cells (**Figures 5H–J**). iNKT1 and iNKT17 cells were also decreased in number in TKO mice compared to WT mice and other control mice (**Figure 5K, M**). We did not observe any changes in iNKT2 cell numbers across all genotypes tested (**Figure 5L**).

We next considered Nur77 expression, and noticed it was significantly reduced in iNKT2 of DKO and TKO when compared to WT cells (**Figure 5N**). Unexpectedly, Nur77 was also reduced in both iNKT2 and iNKT17 cells from RIPK1kd mice as compared to WT controls (**Figures 5O, P**), which implies there may be a kinase-dependent role for RIPK1 in maintaining the Nur77 expression levels in these two subsets. We then measured Ki67 levels as a surrogate for cellular proliferation. iNKT1 and iNKT2 cells from DKO mice expressed lower levels of Ki67, but iNKT17 cells expressed higher levels of Ki67 as compared to WT mice (**Figures 5Q–S**). Therefore, in the absence of RIPK1 (i.e. TKO mice), iNKTp cells accumulate in the thymus and express more RoRγT. This increase in thymic iNKTp frequency also coincides with a parallel increase in the frequency of thymic iNKT2 and iNKT17 cells, which is consistent with a developmental relationship between these populations. TKO mice also reveal that RIPK1 is also required to maintain thymic iNKT1 cell frequency and number. Other changes in thymic frequency and Ki67 expression are more likely to be mediated by Casp8 because they are present in both DKO and TKO mice.

### αGC-induced iNKT cell effector cytokine production may rely on RIPK1/Casp8

**Figure 6:**
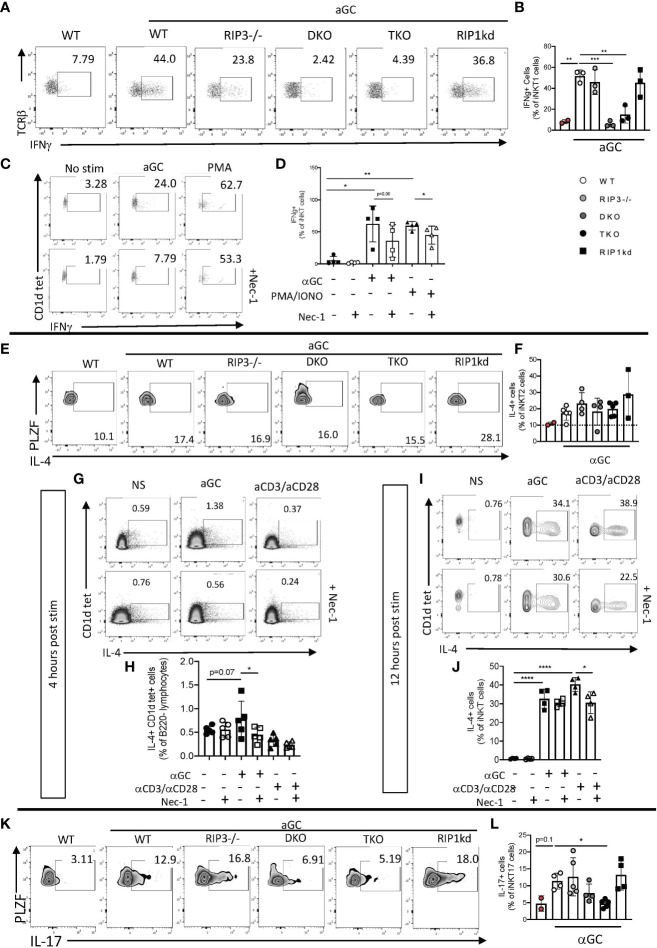
iNKT cell IFNγ, IL-4, and IL-17 effector response to αGC depends on Casp8 and RIPK1. Representative flow cytometry plots identifying frequency of splenic (CD1d tet^+^, TCRβ^int^, PLZF^lo^, RoRγT^-^) iNKT1 cells expressing IFNγ in WT, RIPK3^-/-^, DKO, TKO and RIPK1kd mice four hours after intravenous (i.v)^+^αGC immunization (0.5μg/200μL) **(A)**. Scatterplot quantifies IFNγ^+^ splenic iNKT1 cells **(B)** in indicated mouse strains. Representative flow cytometry plots of eYFP+ (surrogate for IFNγ^+^) splenic iNKT cells from GREAT mice after *in vitro* stimulation with αGC (100ng/ml), PMA/Ionomycin (2μg/ml), and/or Necrostatin1 (39.5μM) for 4 hrs as noted. Scatterplots quantify eYFP^+^ (IFNγ^+^) cells as a frequency of splenic iNKT cells (CD1d tet^+^, TCRβ^int^) **(D)**. Flow cytometry plots depict IL-4^+^ splenic iNKT2 cells (CD1d tet^+^, TCRβ^int^, PLZF^hi^, RoRγT^-^) in all strains 4hrs after i.v. αGC immunization **(E)**. Scatterplot quantifies IL-4^+^ cells from **(E)** as a frequency of splenic iNKT2 cells **(F)**. Representative flow cytometry plots depict hCD2+ (surrogate for IL-4^+^) iNKT cells of *in vitro* stimulated splenocytes from IL-4 reporter (KN2) mice 4 hours **(G)** and 12 hours **(I)** post stimulation with αGC (100ng/ml), αCD3/αCD28 (50μg/ml; 25μg/ml), and/or Necrostatin1 (39.5μM). Scatter plots quantify IL-4^+^ cells as a frequency of iNKT cells 4 hours **(H)** and 12 hours **(J)** after stimulation as indicated. Flow cytometry depicts IL-17^+^ cells as a frequency of splenic iNKT17 cells (CD1d tet^+^, TCRβ^int^, PLZF^int^, RoRγT^+^) **(K)**. Scatterplot quantifies IL-17^+^ cells as a frequency of splenic iNKT17 cells 4hrs after αGC i.v. immmunization **(L)**. Data is representative **(A, C, E, G, I, K)** or a pool **(B, D, F, H, J, L)** of 2-3 independent experiments with at least 2-5 mice per group, 8-16 weeks old. Each symbol indicates an individual mouse. *p ≤ 0.05, **p ≤ 0.01, ***p ≤ 0.001, ****p ≤ 0.0001 (One-way ANOVA).

It is now clear that RIPK1 plays a role in iNKT cell development, but it remained to be determined if RIPK1 also mediates effector functions of peripheral iNKT cells which are induced as a consequence of TcR engagement. To determine if the lack of RIPK1 and/or Casp8 affects iNKT cell cytokine effector function, we immunized WT, RIPK3^-/-^, DKO, TKO and RIPK1kd mice with the iNKT agonist glycolipid αGalactosylceramide (αGC). αGC does not directly activate any cells other than iNKT cells. Four hours after immunization, we analyzed the frequency of splenic IFNγ^+^ iNKT1 cells (gating strategy in **Supplementary Figure 5A**). Immunization with αGC induced an increased frequency of IFNγ ^+^ iNKT cells in WT animals as compared to naive WT animals (**Figures 6A, B**). iNKT cells in αGC-immunized RIPK3^-/-^ and RIPK1kd mice also mounted an IFNγ response comparable to immunized WT mice (**Figures 6A, B**). In contrast, αGC immunized DKO and TKO mice failed to induce IFNγ production by as many of their iNKT1 cells as were induced by immunization of WT mice (**Figures 6A, B**). The shared deficiency in Casp8 between the DKO and TKO mice implicates Casp8 in facilitating the *in vivo* IFNγ^+^production by iNKT1 cells elicited by αGC.

To identify whether a specific blockade of RIPK1 impairs cytokine production in iNKT cells, we *in vitro* stimulated splenocytes from GREAT (IFNγ reporter) mice with αGC alone or in the presence of RIPK1 phosphorylation-inhibitor Necrostatin-1 (Nec-1) (gating strategy in **Supplementary Figure 5B**). PMA/ionomycin served as a positive control stimulus because it induces RIPK1-mediated cytokine production (26). αGC induced IFNγ production by iNKT cells, and Nec-1 pretreatment prevented the increase in IFNγ production by iNKT cells following αGC or control PMA/ionomycin stimulation (**Figures 6C, D**). This suggests that the IFNγ cytokine effector function of iNKT cells depends on a necrostatin-inhibitable kinase, most likely RIPK1.

We next looked at the production of a second effector cytokine, IL-4, another iNKT cell effector cytokine. Upon αGC stimulation, splenic iNKT2 cells rapidly release large quantities of IL-4. To determine if there is a role for RIPK1 in IL-4 production by iNKT cells, we immunized WT mice with αGC and measured the IL-4 response by iNKT cells (gating strategy in **Supplementary Figure 5C**). As expected, αGC induced high levels of IL-4 production by WT iNKT2 cells as compared to naïve WT iNKT cells (**Figures 6E, F**). iNKT2 cells from DKO and TKO mice were equally capable of producing IL-4 as iNKT cells from WT animals (**Figures 6E, F**). In fact, IL-4-producing iNKT cells from all genotypes tested were equally responsive to αGC stimulation (**Figures 6E, F**). To determine if RIPK1 inhibition alone impairs IL-4 production by iNKT cells, we stimulated splenocytes from KN2.Cre mice *in vitro* with αGC or positive control αCD3/αCD28 with/without Nec-1 pretreatment. Two timepoints were needed because RIPK1 inhibition was only evident for αGC induced responses at 4 hour early timepoint (**Figures 6G, H**), but αCD3/αCD28 induced peak IL-4 responses (measured as expression of huCD2 in reporter mice) by 12 hours (**Figures 6I, J**). αGC stimulation induces IL-4 production by iNKT cells *in vitro*, which can be significantly inhibited by *in vitro* pretreatment with Nec-1 (**Figures 6G, H**) (gating strategies in **Supplementary Figure 5D**). αCD3/αCD28 induced robust expression of IL-4 by WT iNKT cells which was significantly inhibited by pretreatment with Nec-1 (**Figures 6I, J**) (gating strategies in **Supplementary Figure 5E**). Therefore, RIPK1 likely plays a role specifically in early αGC-induction of IL-4 *in vitro*.

The production of the proinflammatory cytokine IL-17 is mainly attributed to but not limited to, RoRγT expressing iNKT cells after receptor ligation (27, 28). To consider a possible role for RIPK1 in IL-17 production, we stimulated DKO, TKO, and control mice with αGC and measured intracellular IL-17 using flow cytometry (gating strategies in **Supplementary Figure 5C**). As expected, αGC immunization induced IL-17 production from iNKT17 cells in WT mice (**Figures 6K, L**). αGC immunization of RIPK3^-/-^ and RIPK1kd mice expanded IL-17^+^ iNKT17 frequency to the same increased level as WT immunized mice (**Figures 6K, L**). However, iNKT cells from αGC immunized DKO and TKO mice had lower frequency of IL-17^+^ iNKT17 cells when compared to WT immunized mice (**Figures 6K, L**), although only the difference in the TKO mice was statistically significant. This is consistent with IL-17 cytokine effector function depending on Casp8/RIPK1. Given the *in vivo* evidence for Casp8-dependence of IFNγ and IL-17 responses to iNKT activation, and the *in vitro* evidence for Casp8/RIPK1-dependence of IFNγ and IL-4 responses to iNKT activation, it is most likely that iNKT cell effector function in response to αGC generally requires both Casp8 and RIPK1.

### iNKT_FH_ cell expansion is RIPK1/Casp8 independent

**Figure 7:**
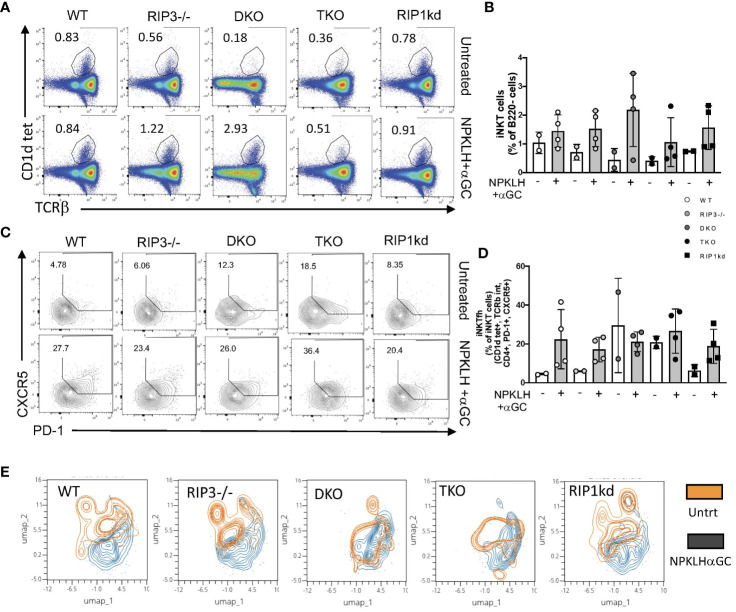
DKO and TKO mice develop increased iNKT_FH_ frequency in naïve animals. Representative flow cytometry plots capture iNKT cell (CD1d tet^+^, TCRβ^int^) frequencies in untreated (top) and NP-αGC (0.5μg/200μL; bottom) immunized WT, RIPK3^-/-^, DKO, TKO and RIPK1kd mice **(A)**. Scatter plot quantifies iNKT cells as a frequency of B220^-^ lymphocytes from mice in **(A)** as indicated **(B)**. Representative flow plots capture CD4+ iNKT_FH_ cells (CD1d tet^+^, TCRβ^int^, CD4^+^, CXCR5^+^, PD-1^+^) in untreated (top) and NPαGC immunized (bottom) WT, RIPK3^-/-^, DKO, TKO and RIPK1kd mice **(C)**. Scatter plot quantifies iNKT_FH_ cells as a frequency of CD4^+^ iNKT cells from mice in **(C)** as indicated **(D)**. UMAP plots depict iNKT_FH_ (CD1d tet^+^, TCRβ^int^, CD4^+^, PD-1^+^, CXCR5^+^) populations in untreated (orange) or NPαGC immunized (black) mice based on MFI of PD-1, Tim3, Lag3, CXCR5, and ICOS **(E)**. Data is representative **(A,C,E)** or a pool **(B,D)** of 2 independent experiments with 2-4 mice per group, 8-16 weeks old. Each symbol indicates an individual mouse.

To extend these studies to consider the role of Casp8 and RIPK1 in mediating another iNKT effector function, we looked at the induced expansion of iNKT_FH_ cells. We have previously demonstrated that immunization of mice with NP-KLH plus αGC activates iNKT cells to express CXCR5, bcl-6, and PD-1, rending them capable of providing helper signals to activated B cells (29). To determine if RIPK1 is required for innate iNKT_FH_ cell expansion, we immunized mice with NP-KLH plus αGC. Seven days after immunization we used flow cytometry to measure the frequency of splenic iNKT cells and iNKT_FH_ cells from immunized WT, RIPK3^-/-^, DKO, TKO and RIPK1kd mice and their naïve counterparts (gating strategy in **Supplementary Figure 6**). NP-KLH plus αGC immunized animals had only a modestly higher frequency of TcRβ+ CD1d-tet+ iNKT cells compared to their respective naïve controls (**Figures 7A, B**). NP-KLH plus αGC immunization expanded iNKT_FH_ cells in WT, RIPK3^-/-^, and RIPK1kd mice compared to their respective naïve controls, but because of large variability and high iNKT_FH_ background frequencies, did not show any increases in iNKT_FH_ cells in DKO and TKO mice (**Figures 7C, D**).

To assess the activation/exhaustion status of iNKT_FH_ cells in mice lacking RIPK1/Casp8 in response to NP-KLH and αGC, we measured expression of a combination of exhaustion and activation markers; PD-1, Lag3, Tim3 and ICOS. We used UMAPs to visualize naïve and activated populations of iNKT_FH_ cells in an unbiased format across all genotypes of mice. The Umap analysis considers the surface expression by MFI of PD-1, CXCR5, ICOS, Tim3 and Lag3 proteins, and then reveals populations of cells which cluster together as a consequence of similar marker expression. The Umap analysis identified clear populations of naive and activated iNKT_FH_ cells in WT, RIPK3-/- and RIPK1kd mice. However, in DKO and TKO mice, the naïve iNKT_FH_ population overlapped extensively with the activated iNKT_FH_ population (**Figure 7E**), suggesting these iNKT_FH_ cells may already exist in the naïve DKO and TKO mice. This is consistent with the increased frequencies of iNKT_FH_ cells observed in the un-immunized mice (**Figures 7C, D**). In summary, iNKT_FH_ cell expansion in response to NP-KLH plus αGC is independent of RIPK1/Casp8. Naïve DKO and TKO mice may harbor preactivated iNKT_FH_ cells in their spleens consistent with an ongoing autoimmune response because of their shared lack of Casp8.

## Discussion

RIPK1 works with Casp8 to orchestrate programmed cell death. Apoptosis, a form of programmed cell death, is crucial for T cell development. Apoptosis eliminates T cells that are autoreactive or faulty in order to prevent autoimmunity. Loss of function mutations preventing kinase activity of RIPK1 itself as well as heterozygous Casp8 cleavage-site mutations allowing unregulated activity of RIPK1 cause severe primary immunodeficiency, intestinal inflammation, arthritis, and CRIA in humans (1, 30). RIPK1 also plays an important role in activating the NFκB pathway to promote release of inflammatory cytokines such as IL-1β and TNFα by endothelial cells and macrophages (31). Studying the role of RIPK1 in mediating inflammation using mouse models is complicated by the fact that RIPK1 deficient mice die soon after birth. Similarly, Casp8 deficient mice die during embryogenesis as a result of excessive vascular and cardiac defects. However, knocking out both RIPK3 and Casp8 (DKO) avoids embryonic lethality (4). Mice triply deficient for RIPK1, RIPK3 and Casp8 (TKO) also survive well into adulthood. To study the *in vivo* role of RIPK1, we compared the leukocyte populations and their phenotypes in TKO and DKO mice to RIPK3^-/-^ and WT mouse controls. In DKO mice, a deficiency of Casp8 removes RIPK1 regulation, so RIPK1 is unfettered and is likely to be more active (4, 10). Conversely, in TKO mice, both RIPK1 and the negative regulation are missing, so RIPK1 activity is more likely to be absent (32). Therefore, the TKO mice closely reflect the autoimmune phenotype in humans caused by loss of function mutations in RIPK1, while the DKO mice better reflect the genotype found in humans carrying a mutation rendering RIPK1 constitutively active (CRIA patients).

The absence of RIPK1 and Casp8 leads iNKT cells to be present at a lower frequency and number in the periphery, suggesting a previously undefined role for RIPK1 in maintaining innate T cell homeostasis. Neither MAIT or γδT cell numbers are consistently affected by a RIPK1 deficient environment. Thus, the RIPK1/Casp8 regulation is unique to iNKT cells. Specifically, the role of Casp8, determined based on changes detected in both Casp8-deficient DKO and TKO mice, was found to be key for peripheral innate T cell homeostasis and effector function. In both Casp8 deficient (DKO, TKO) environments, total splenic iNKT cell numbers and frequencies are reduced. Within that reduced population in DKO mice, iNKT2 cells trend towards being underrepresented with a slight concomitant overrepresentation of iNKT1 cells. Thus, it appears that Casp8 supports healthy iNKT cell numbers and maintains peripheral iNKT subset distribution.

The specific contribution for RIPK1, determined by referencing differences between DKO and TKO mice, reveals a more centralized role restricted primarily to the thymus. For example, iNKT cells do not progress from stage 2 (CD44^+^, NK1.1^-^) to stage 3 (CD44^+^, NK1.1^+^) of development in the thymus, and NK1.1 protein expression is never induced. There are two well established control points in iNKT development. Control point 1 directs CD1d mediated iNKT cell selection into stage 0 or 1, which occurs only in the thymus. Control point 2 directs iNKT cell maturation from stage 2 to stage 3, and is defined by NK1.1 upregulation. This can occur in the thymus or the periphery (33). In accordance with these control points, our data suggests that RIPK1 mediates iNKT progression through control point 2 by facilitating NK1.1 protein expression. Because we are distinguishing iNKT cell stages 2 and 3 primarily by expression of CD44 and NK1.1, there is a possibility that isolated loss the NK1.1 protein could result in a hybrid population which is a variant of the iNKT stage 3 cell lacking NK1.1, but this population is unlikely to be a physiologically translational population since it is only a consequence of our artificial interruption of RIPK1, so we have not characterized this population further. This developmental checkpoint may be generalizable to other cells, since NK1.1 protein expression on NK cells is also compromised in the absence of RIPK1 which might render them non-functional (34). The role of RIPK1 in NK cell populations warrants further investigation. Further study is also needed to assess whether the requirement for RIPK1 to mediate NK1.1 protein expression is cell intrinsic or cell extrinsic. This has not been specifically addressed in these studies, but a mixed bone marrow chimeric approach or an iNKT or NK cell lineage-specific deletion of RIPK1 would be useful to resolve this remaining question.

DKO mice lack casp8, thus removing the brakes that regulate RIPK1 activity, and creating an environment with uninhibited RIPK1 activity. However, excessive RIPK1 activity has no effect on iNKT thymic development. Contrastingly, the complete absence of RIPK1 in TKO mice disrupts iNKT development. This effect is not observed in RIPK1kd mice which have an inactive kinase domain, which implicates a role for the scaffolding domain of RIPK1 in mediating iNKT development. Our previous study described a potential role for the RIPK1 scaffolding domain in bridging signals between the B cell receptor (BcR) and NFκB signaling molecules (11), and these iNKT data suggest a similar mechanism might be at play downstream of the TcR. Strikingly, the collective observations from our investigations highlight a role of RIPK and Casp8 signaling pathways regulating a particularly innate-type lymphocyte development and function.

Without RIPK1, iNKTp cells accumulate in the thymus of TKO mice, leading to an increase in percentage of iNKTp within the entire iNKT pool. Of the iNKTp cells that do mature in the TKO thymus, fewer of them acquire the iNKT1 phenotype than in other control mice, while the rest mature into frequencies of iNKT2 and iNKT17 cells higher than controls. This is consistent with the linear model of thymic iNKT cell development (35). In the linear model, levels of TcR surface expression and CD1d-TcR avidity correlate with iNKT cell subset differentiation, where highest to lowest TcR binding drives iNKT2 > iNKT1 > iNKT1 development (36, 37). In previous studies, mouse strains with mutations in ZAP70 or Egr2 had lower TcR signaling thresholds and the mice develop more iNKT1 cells and fewer iNKT2 or iNKT17 cells than WT controls (36, 37). Consistent with these models, we see modestly increased iNKT2 and iNKT17 cells and fewer iNKT1 cells in TKO mice lacking RIPK1, RIPK3, and Casp8, which is consistent with a loss of signal amplification by RIPK1 downstream of the TcR.

TKO phenotype includes a dominant presence of stage 1 and 2 cells (which differentiate into iNKT2 and iNKT17 cells), and a reduced frequency of stage 3 cells (which differentiate into iNKT1 cells) (24). Progression of stage 2 to stage 3 iNKT cells as well as differentiation of iNKTp cells requires signaling through the TcR to acquire other fate defining features such as NK1.1 expression (38). As noted above, in the absence of RIPK1, we observed reduced iNKT1 frequency in the thymus which is consistent with reduced signal transduction through the TcR. Signal through the TcR is required for stage 2 iNKT cells to acquire NK1.1 expression on their surface which allows progression to stage 3 and eventual development into iNKT1 cells. Therefore, we propose that RIPK1 acts downstream of the TcR to regulate expression of other co-factors required for iNKT1 maturation and stage 2 to stage 3 iNKT cell progression. These results suggest a model whereby RIPK1 may participate in conveying signals between the TcR and NFκB, which then translates these signals to the nucleus and engages the promoter of *Nkrp1* to drive NK1.1 expression (**Supplementary Figure 7**). Previous studies have dissected possible kinase-dependent roles for RIPK1, RIPK3, and Casp8 in mediating cell death during conventional T development in the thymus [summarized in (39)], but the intricacies of a scaffolding-dependent signaling cascade in iNKT cells is beyond the scope of these studies.

Further *in vivo* analysis revealed that splenic iNKT cells from DKO and TKO mice, but not RIPK3 deficient mice, had impaired inflammatory cytokine production in response to iNKT agonist αGC, which we attributed to the shared absence of Casp8. Previous studies did find a role for RIPK3 in mediating IL-4, TNFα, and IFNγ by liver iNKT cells via PGAM5, Drp1, and NFAT, but those studies were performed with liver iNKT cells, while we studied splenic iNKT cells, so that may explain some of the discrepancy between published studies and our findings here (40). Similarly, we found that RIPK1 blockade alone with necrostatin in an *in vitro* setting also reduced inflammatory cytokine production by splenic iNKT cells, but this was not observed in liver iNKT cells (40). Recent studies connected T cell-restricted RIPK1 deficiency to higher basal levels of mTOR activation and increased senescence of conventional T cells which could be restored to healthy levels by RIPK3 and Casp8 deficiency/blocking or a transfer to a normal *in vivo* environment (41). These studies show that RIPK1 mediates conventional T cell intrinsic effects, in some cases through RIPK3/Casp8, but as we also see with iNKT cells, those effects are likely to be tuned by environmental signals including TCR signals, costimulatory signals, growth factors, or nutrients (41).

Surprisingly, RIPK1kd mice exhibited an iNKT cell phenotype which resembles WT mice in many ways. This suggests that the iNKT cell phenotype present in TKO mice is most likely not a consequence of the loss of the RIPK1 kinase domain, but rather the scaffolding domain. The gain-of-function mutations responsible for the autoimmune phenotype observed in human autoimmune CRIA lie in the loss of a RIPK3 cleavage site in the scaffolding domain of the RIPK1 protein. Our results also implicate the scaffold domain of the RIPK1 protein in regulating the development of iNKT cells. Injection with NP-KLH and αGC (not shown) and NPαGC did not affect the expansion of total iNKT cells or iNKT_FH_ cells in DKO and TKO mice suggesting no role for RIPK1/Casp8 in iNKT cell effector cell expansion following immunization. However, hyperactivated iNKT_FH_ cells were present on a RIPK1/Casp8 background, consistent with the autoimmune phenotype in those mouse strains.

In summary, our results indicate a supporting role for RIPK1 in orchestrating central innate T cell development. We find that RIPK1 is required for iNKT cells to progress through the second control point in the linear model of development. Similarly, iNKTp cells fail to progress into NK1.1-expressing mature iNKT cells on a RIPK1 deficient background. If RIPK1 deficiency produces interruptions downstream of the TcR, this could also explain the reduction in inflammatory cytokine production by iNKT cells from TKO mice. Thus, our study reinforces previous evidence for RIPK1 activity downstream of the TcR and suggests it may act as a rheostat in conveying or amplifying the strength of TcR signal. Furthermore, the present study raises the possibility of using small molecule inhibitors of RIPK1 to potentially regulate iNKT cell expansion and cytokine responses to alleviate autoinflammatory conditions in humans.

## Data availability statement

The raw data supporting the conclusions of this article will be made available by the authors, without undue reservation.

## Ethics statement

The animal study was approved by UTHSCSA Animal Care and Use Committee (ACUC). The study was conducted in accordance with the local legislation and institutional requirements.

## Author contributions

TH, RP, and EL conceived and designed the study. RP, TH, NL, ED and EL acquired and/or interpreted data. TH, RP, and EL wrote the article. All authors contributed to the article and approved the submitted version.
