# Supplementary material for: RIPK1 deficiency prevents thymic NK1.1 expression and subsequent iNKT cell development
Source: Front Immunol. 2023 Oct 30;14:1103591. doi: 10.3389/fimmu.2023.1103591 (PMC10642909; doi:10.3389/fimmu.2023.1103591)
Supplement: Supplementary file 1 [file Presentation_1.pdf]

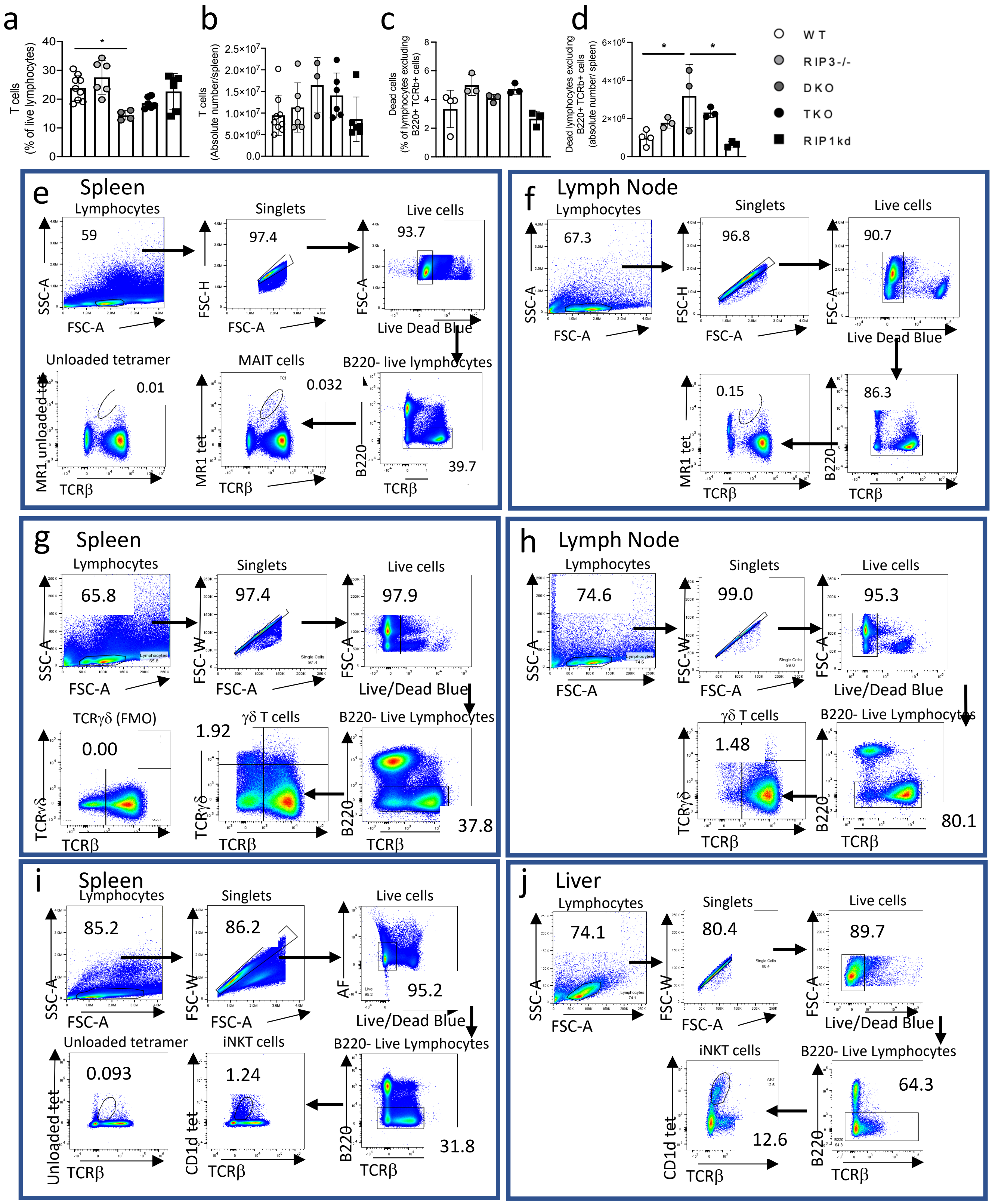

Supplemental Figure 1. DKO mice have reduced T cells and increased dead lymphocytes. Scatterplot quantifies the frequency **(a)** and absolute number **(b)** of total TCRβ$^+$ splenic T cells as a subset of live lymphocytes in WT, RIP3$^{-/-}$, DKO, TKO and RIP1kd mice. Scatter plot of frequency **(c)** and number **(d)** of dead lymphocytes staining positive for viability dye in the spleens of mice from **(a)** as indicated. Flow cytometry shows gating strategies for B220-TcRβ+MR1 tet+ MAIT cells in spleen **(e)** and lymph node **(f)**, B220-TcRβ-TcRγδ+ γδT cells in spleen **(g)** and lymph node **(h)**, as well as B220-TcRβ+CD1d tet+ iNKT cells in spleen **(i)** and liver **(j)**. Data is a pool **(a-c)** of 3-4 independent experiments with 2-6 mice per group or representative flow plots from same, 8-16 weeks old. Each symbol indicates an individual mouse. *p≤0.05, **p≤0.01, ***p≤0.001, ****p≤0.0001 (One-way ANOVA).

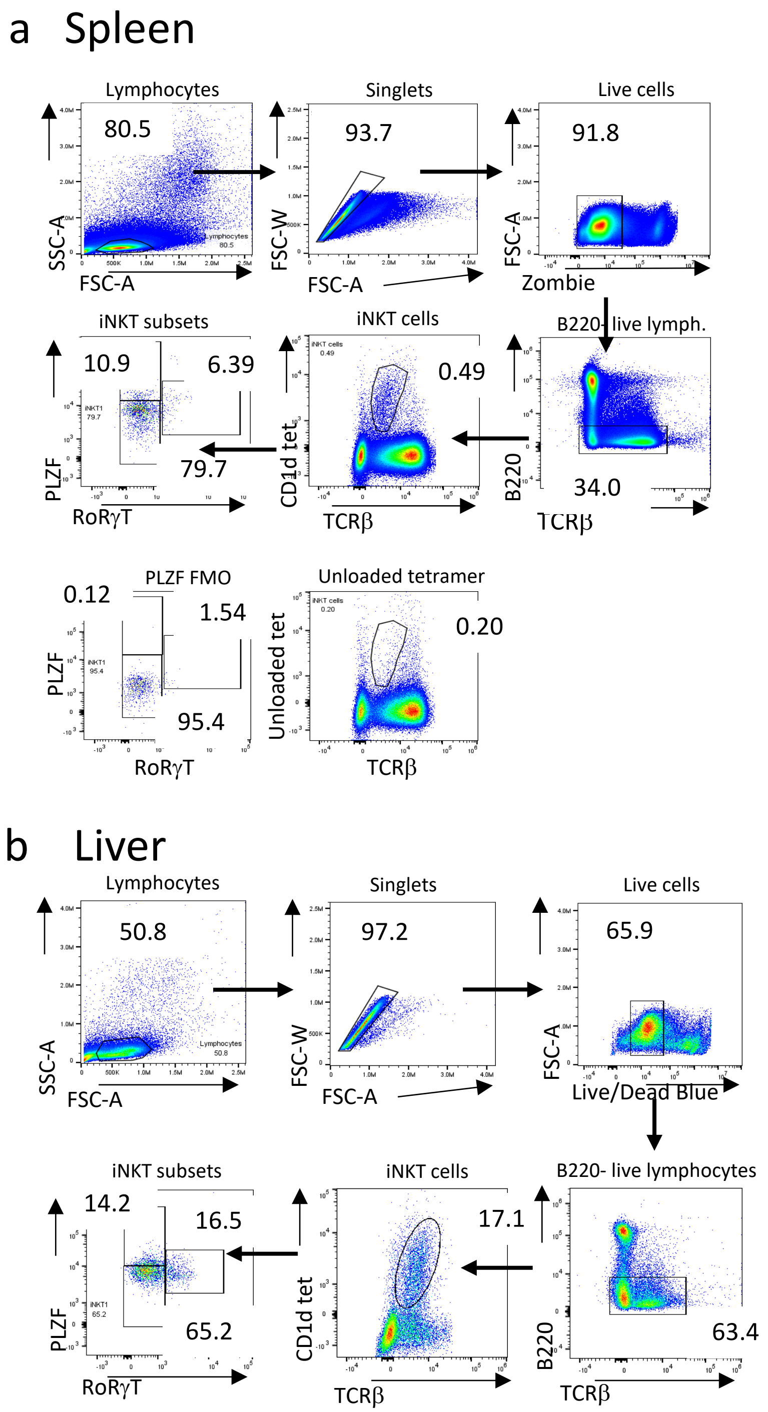

Supplemental Figure 2. Flow cytometry gating strategies for iNKT cell subsets in spleen and liver. Flow cytometry shows gating strategies for RoRγT- PLZF- iNKT1, RoRγT- PLZF+ iNKT2, and RoRγT+ PLZF^{lo} iNKT17 subsets from representative B220-TcRβ+CD1d tet+ iNKT cell population in spleen **(a)** and liver **(b)**. PLZF FMO and unloaded tetramer controls included for comparison.

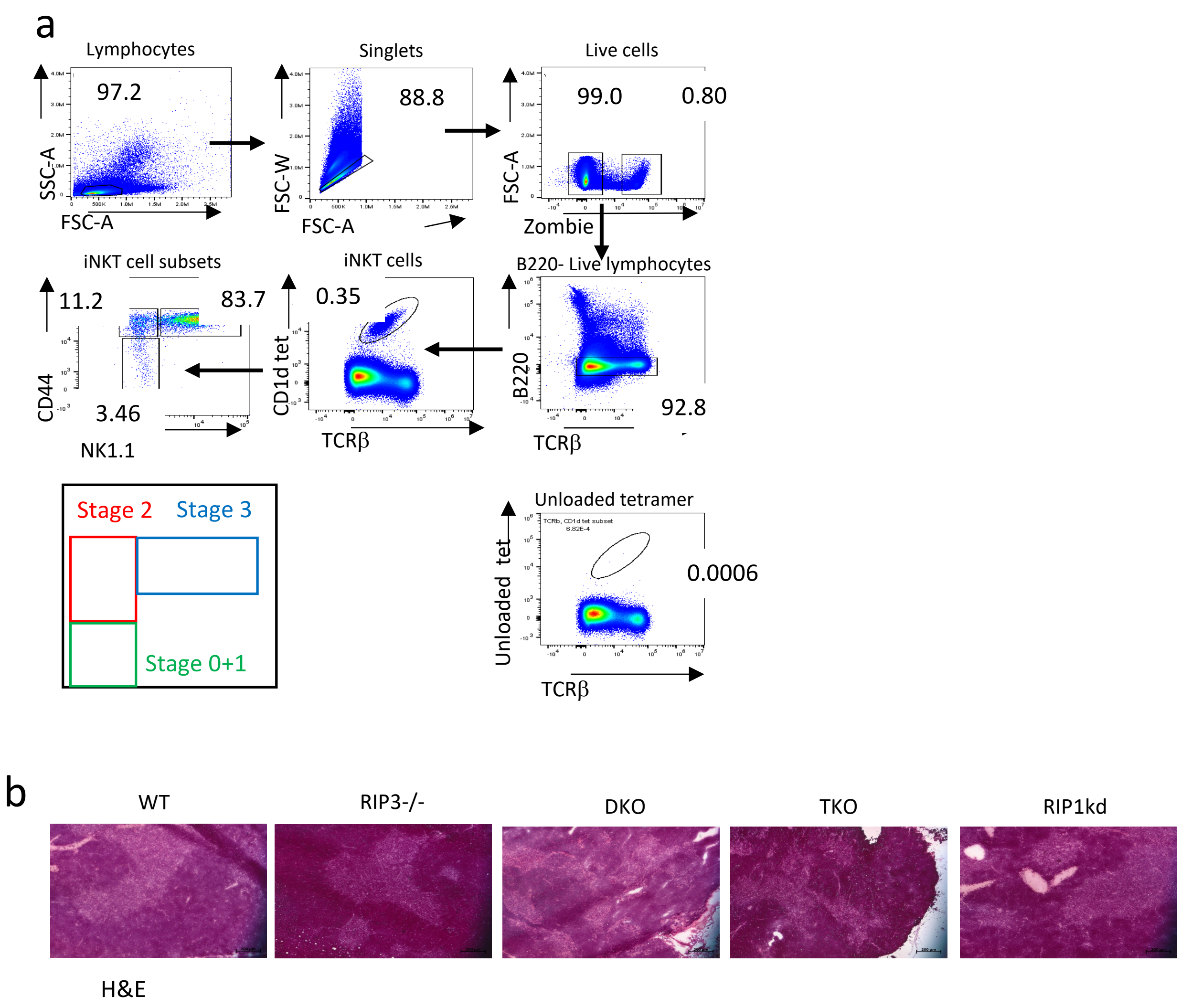

Supplemental Figure 3. Flow cytometry gating strategies for iNKT cell stages in thymus. Flow cytometry shows gating strategies for NK1.1- CD44- iNKT stage 0+1, NK1.1- CD44+ iNKT stage 2, and NK1.1+ CD44+ iNKT stage 3 subsets from representative B220-TcRβ+CD1d tet+ iNKT cell population in thymus **(a)**. Unloaded tetramer control included for comparison. H&E images of 6mm thick sections of thymi of mice as indicated **(b)**.

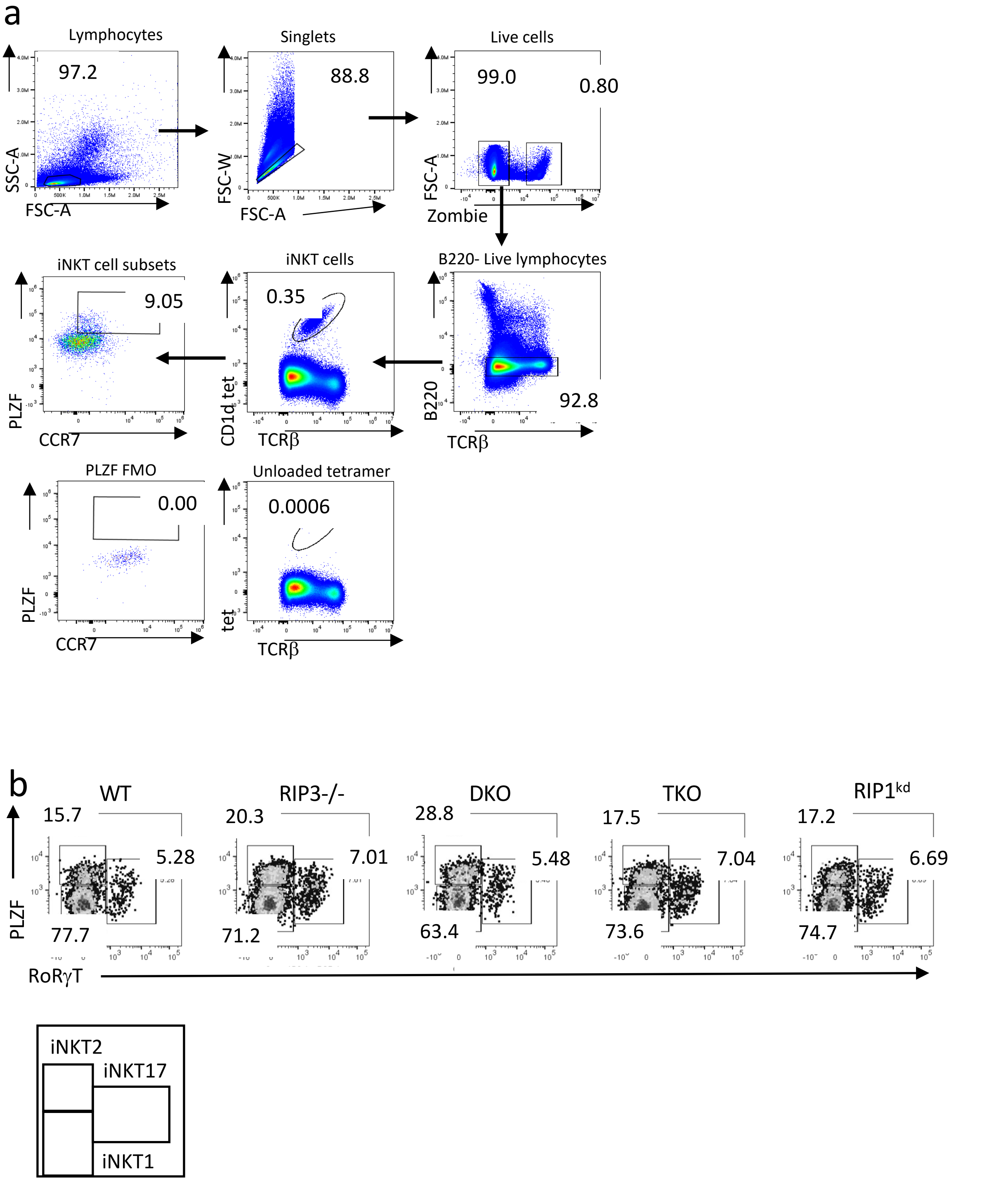

Supplemental Figure 4. Flow cytometry gating strategies for iNKT cell precursors and iNKT1, 2, and 17 subsets in thymus. Flow cytometry shows gating strategies for PLZF+ CCR7+ iNKT precursors from representative B220-TcRβ+CD1d tet+ iNKT cell population in thymus **(a)**. PLZF FMO and unloaded tetramer controls included for comparison. Representative RoRγT- PLZF- iNKT1, RoRγT- PLZF+ iNKT2, and RoRγT+ PLZF^{lo} iNKT17 subset frequencies from B220-TcRβ+CD1d tet+ iNKT cell population in thymus **(b)**.

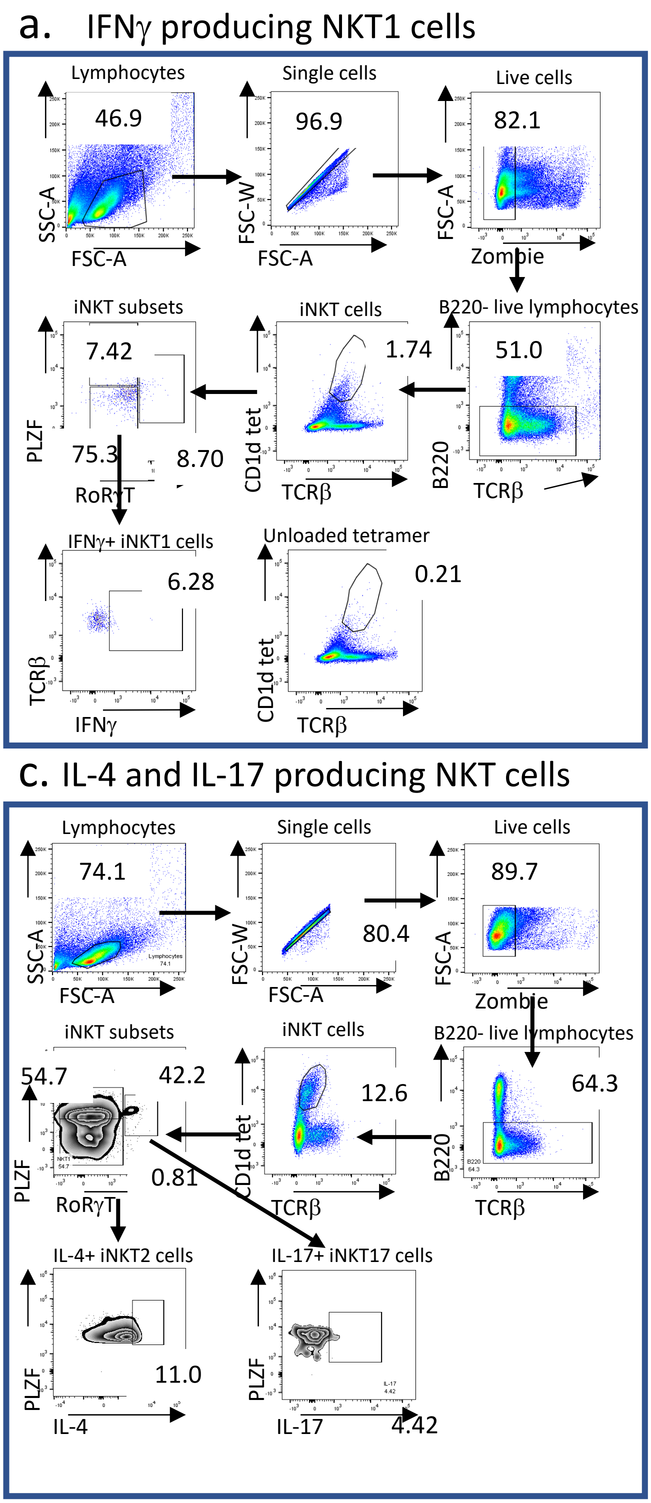

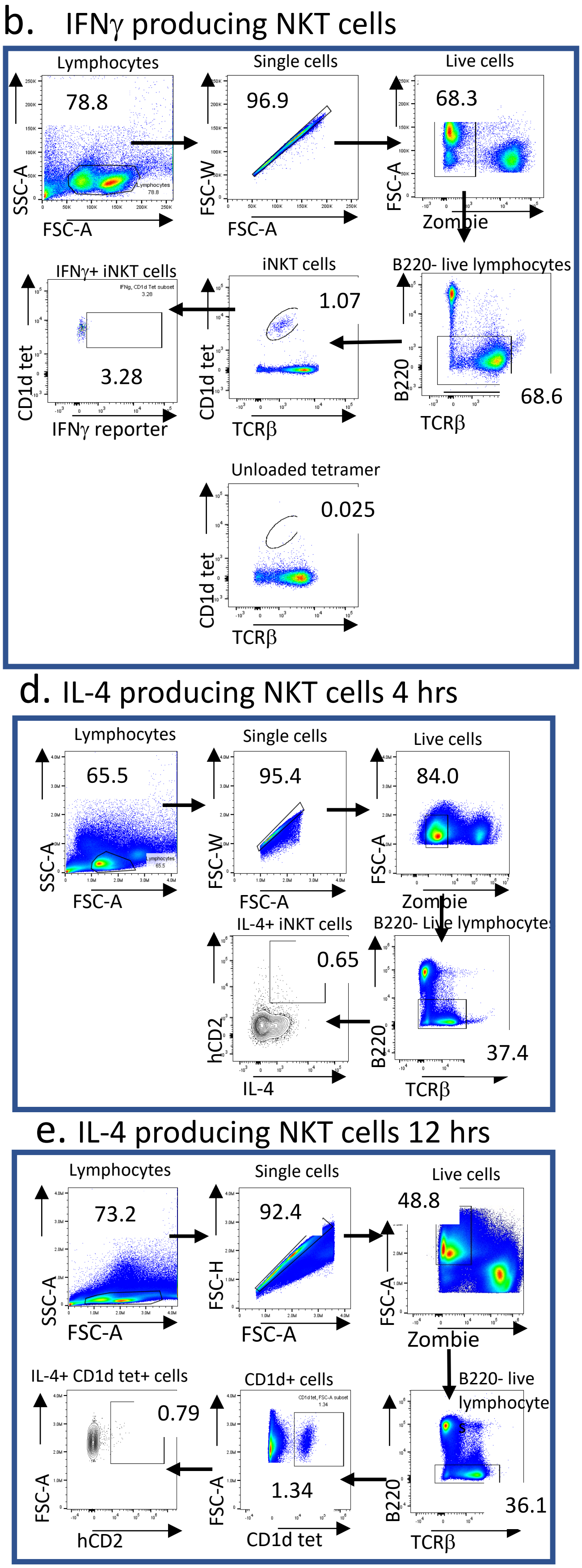

Supplemental Figure 5. Flow cytometry gating strategies for IFNγ, IL-4, and IL-17 producing iNKT cells in splenocytes. Flow cytometry shows gating strategies for IFNγ producing PLZF+ RoRγT- iNKT1 cells from B220-TcRβ+CD1d tet+ iNKT cell population in spleen **(a)**. Corresponds to data in **Figure 6a,b**. Gating strategies for (eYFP+) IFNγ producing B220-TcRβ+CD1d tet+ iNKT cells in spleen of GREAT reporter mouse **(b)**. Corresponds to data in **Figure 6c,d**.

Gating strategies for RoRγT-PLZF+ IL4-producing iNKT2 and RoRγT+PLZF^{lo} IL-17 producing iNKT17 cells using sub-gated B220-TcRβ+CD1d tet+ iNKT cells **(c)**. Corresponds to data in **Figure 6e,f** (IL-4) and **Figure 6k,l** (IL-17). Gating strategies for (huCD2+) IL-4 producing B220-TcRβ+CD1d tet+ iNKT cells in spleen of B6.KN2 reporter mouse after in vitro stimulation for 4hrs **(d)** or 12 hrs **(e)**. Corresponds to data in **Figure 6g and l**, respectively. Unloaded tetramer controls included for comparison.

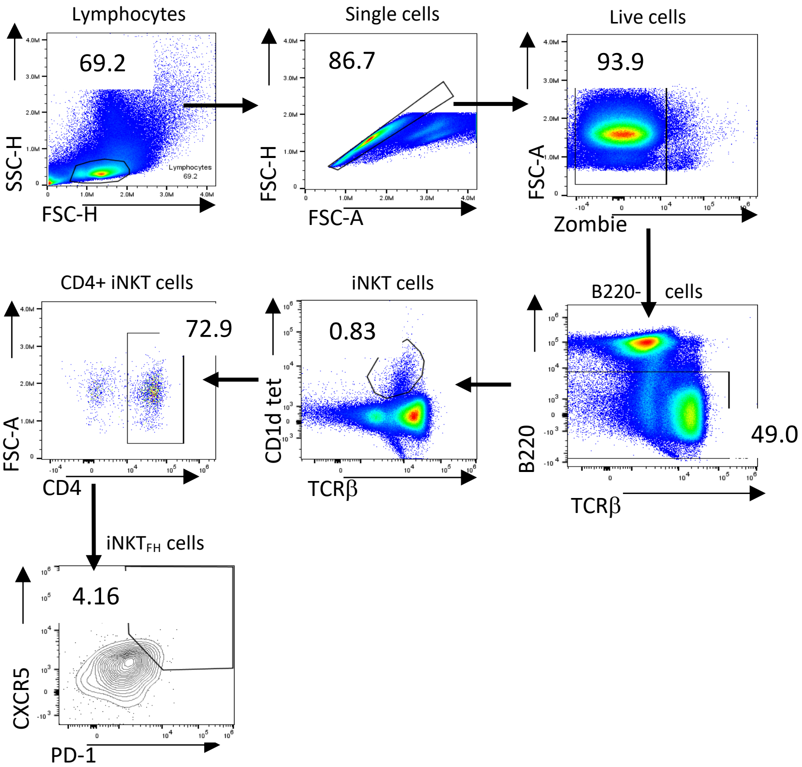

Supplemental Figure 6. Flow cytometry gating strategies for iNKT$_{FH}$ cells in spleen. Flow cytometry shows gating strategies for CXCR5+ PD-1+ iNKT$_{FH}$ cells from representative B220-TcRβ+CD1d tet+CD4+ iNKT cell population in spleen.

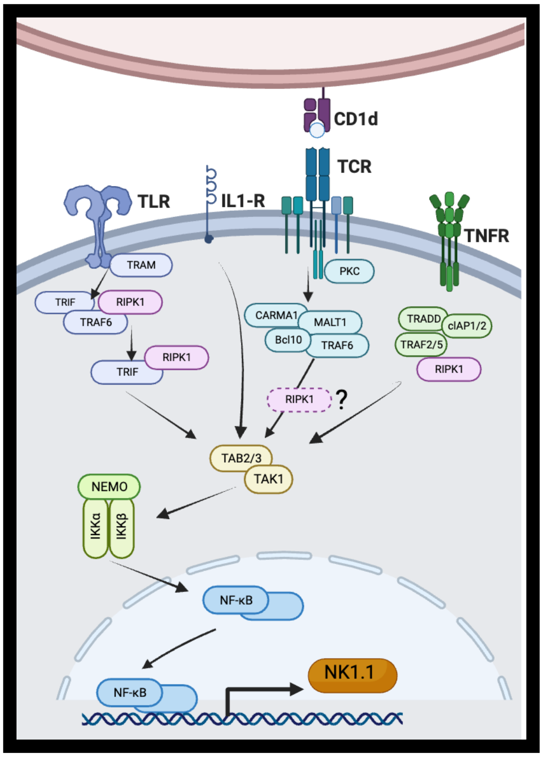

Supplemental Figure 7. Proposed model for role of RIPK1 between TcR engagement and NFκB-driven expression of NK1.1. Created by Biorender.

Sl No	Antibody	Fluorophore	Clone	Catalog
1	CCR7	PE	4B12	560682
		BV421	4B12	BDB566291
2	CD1d	BV421	1B1	562712
		Pac Blue	1B1	123517
		AF647	1B1	123512
		PerCP Cy5.5	1B1	562713
3	CD4	AF405	RM4-5	MCD0426
		BV650	RM4-5	100545
		FITC	RM4-5	553047
		FITC	GK 1.5	11-0041-82
		PE	RM4-5	12-0042-82
		PE-TexRed	RM4-5	MCD0417
		PerCP Cy5.5	GK 1.5	100433
		PerCP	RM4-5	553052
		PE-Cy7	RM4-5	100528
		APC	RM4-5	100516
		APC-Cy7	RM4-5	47-0042-82
		eFluor615	RM4-5	50-245-793
4	CD44	BV410	IM7	103043
		PE	IM7	553134
		PE Cy5	IM7	553135
		APC	IM7	17-0441-81
		APC-Cy7	IM7	47-0441-82
5	CD49b	BV421	DX5	563063
		Pacific Blue	DX5	108917
		FITC	DX5	11-5971-81
		PE-Cy7	DX5	108921
		FITC	DX5	50-993-5
		eFluor506	DX5	69-5971-80
6	CD69	BV421	H1 2F3	104527
		FITC	H1.2F3	104506
		PE	H1.2F3	553237
		Per Cp-Cy5.5	H1.2F3	551113
		PE-Cy7	H1.2F3	25-0691-82
		APC-Cy7	H1.2F3	104526
7	CD8	Pacific Blue	53-6.7	100725
		Pacific Orange	5H10	MCD0830
		Alexa Fluor 405	5H10	MCD0826
		FITC	2.43	35-1886-U025
		PE	53-6.7	553033
		PE	53-6.7	12-0081-82
		Per Cp	53-6.7	553036
		PE-Cy7	5H10	MCD0812
		APC-AF750	53-6.7	27-0081-82
		APC	53-6.7	17-0081-82
		Alexa Fluor 700	53-6.7	557959
		APC-Cy7	53-6.7	100714
		BUV395	53-6.7	563786
8	CXCR5	PE	2G8	551959
		Biotin	2G8	551960
9	hCD2	Biotin	RPA-2.10	555325
		PE	RPA-2.10	555327
10	ICOS	PE	C398.4A	12-9949-81
		APC	C398.4A	313510
11	IFNg	Pacific Blue	XMG 1.2	57-7311-82
		PE	XMG 1.2	554412
		Per Cp-Cy5.5	XMG 1.2	505822
		APC	XMG 1.2	554413
		Alexa Fluor 647	XMG1.2	557735
		PE-Cy5	XMG1.2	55-7311-SU05
		PE-Cy5	XMG1.2	55-7311-U025
12	IgG1	BV605	A85-1	563285
		FITC	A85-1	553443
		PE	A85-1	550083
13	IL-17A	BV650	TC11-18H10.1	506929
		PE	11B11	12-7041-82
14	IL-4	APC	11B11	554436
		APC	11B11	17-7041-81
15	Ki67	Per Cp-Cy5.5	B56	561284
16	Lag3	PE	C9B7W	125207
17	NK1.1	BV650	PK136	108736
		PE	PK136	553165/557391
		APC-Cy7	PK136	560618
18	Nur77	PerCP-eFluor710	12.14	46-5965-82
19	PD-1	PE	J43	551892
		BUV737	RMP1-30	749306
20	PLZF		D-9	SC-28319
21	RORgT	PE	Q31-378	562607
		AF647	Q31-378	562682
		Per Cp-Cy5.5	Q31-378	562683
22	Tbet	BV785	4B10	644835
		PE-Cy7	4B10	644824
23	TCRb	Pacific Blue	H57-597	109226
		BV510	H57-597	109234
		BV605	H57-597	109241
		BV650	H57-597	742483
		FITC	H57-597	109206
		PE	H57-597	553172
		Per Cp-Cy5.5	H57-597	109227
		APC	H57-597	17-5961-83
		APC/Cy7	H57-597	109220
24	TCRgd	PE		HM3804
		APC	eBioGL3	17-5711-81 or 82
25	Tim3	APC	B8.2C12	134007
		BV711	B8.2C12	134021
26	B220	BV785	RA3-6B2	103246

Supplementary Table 1. Flow cytometry antibodies used in these studies.

Gene of interest	Forward Primer	Reverse Primer
NKRP1a	5’ GCACAATGGACACAGCAA 3’	5’ GTAGACATGGCTCAGTGATTG 3’
NKRP1B	5’ CAATGGATTCAACAACACTGGTC 3’	5’ GGACAGGGGAGAGATGGAGAT 3’
NKRP1C	5’ TGAAATGGACACAGCAAGTATC 3’	5 GAGTCAACGAATGGAAAGGAA 3’

Supplementary Table 2. PcR primers used for NK1.1 alleles.
